# Ultrasensitive Enzyme-Free Detection of Proteins on Magnetic Beads

**DOI:** 10.1021/jacs.5c19560

**Published:** 2026-03-13

**Authors:** Karan Malhotra, Louise L. Hansen, Samuel Han, Shira Roth, David R. Walt

**Affiliations:** † Department of Pathology, Brigham and Women’s Hospital, Harvard Medical School, Boston, Massachusetts 02115, United States; ‡ Wyss Institute for Biologically Inspired Engineering, 1812 Harvard University, Boston, Massachusetts 02215, United States; § Harvard Medical School, 1812 Harvard University, Boston, Massachusetts 02115, United States

## Abstract

Ultrasensitive measurements of low abundance protein biomarkers from minimally invasive biofluids (*e.g*., plasma or urine) can enable detection of diseases and transform clinical diagnostics and treatment. Digital protein detection assays, such as Single Molecule Arrays (Simoa) and Molecular On-bead Signal Amplification for Individual Counting (MOSAIC), enable ultrasensitive protein quantification, achieving detection limits that are at least 1000-fold lower than those of conventional ELISA assays. Currently, these ultrasensitive assays are performed in centralized laboratories that have access to specialized instrumentation and stable supply chains, which are rarely available in low- and middle-income countries (LMIC) or at the point-of-care (POC) in resource-limited settings. To address the need for ultrasensitive, accessible diagnostic assays, we have developed an enzyme-free platform for the detection of protein biomarkers with simple assay workflows. This approach uses enzyme-free signal amplifiers generated using hybridization chain reaction to achieve detection limits comparable to Simoa for most proteins. The signal amplifier reagent retains full performance when stored in the dark at 21 °C for up to six months. To investigate the utility of efMOSAIC for clinical workflows, we validated the platform for the sensitive detection of cytokines from human plasma and demonstrated multiplexed biomarker detection. With simple assay workflows and temperature stable reagents, efMOSAIC is poised to transform ultrasensitive protein assays making these technologies more accessible for use in LMICs.

## Graphical abstract

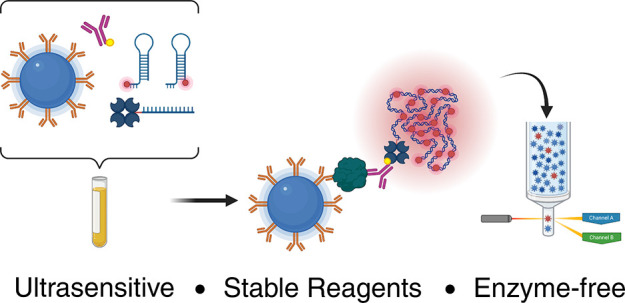

## Introduction

There is an urgent need for accessible diagnostics technology to help alleviate the global health burden of disease. Due to limited access to testing, many patients in low- and middle-income countries (LMICs) remain untreated for diseases, leading to increased morbidity and mortality.^1^ For example, patients infected with *Mycobacterium tuberculosis (M.tb)* can be cured with antibiotics in six months to two years. However, many never begin treatment and remain undiagnosed due to lack of accessible testing in LMICs.^1,2^ As a result, mortality from infectious diseases remains highest in these countries, underscoring the urgent need for improved diagnostics testing.^1,3^ Furthermore, patients with cancers (*e.g*., prostate, ovarian, or breast cancer)^4^ or neurodegenerative diseases (*e.g*., Alzheimer’s disease)^5,6^ are often diagnosed when they become symptomatic at a later disease stage in LMICs, when there are less treatment options. Diagnosing these patients earlier when they are asymptomatic would allow for more treatment options and better clinical outcomes.

The most commonly used methods for diagnosing infectious diseases (*e.g*., culture testing or nucleic acid amplification tests) rely on detecting pathogenic material from patient samples.^1,3^ In the case of Tuberculosis, trained healthcare workers process highly infectious sputum samples in specialized clinical laboratories, which limits accessibility and raises the cost for testing.^3,7^ Early detection of cancers and neurodegenerative diseases remains challenging because patients must undergo specialized testing (*e.g*., surgical biopsies, cerebrospinal fluid testing, or positron emission tomography scans) in well-equipped laboratories that are not always available in many communities.^6,8,9^ One promising alternative to these diagnostic workflows is the measurement of protein biomarkers in minimally invasive biofluids such as plasma, serum, or urine.^10,11^ Measuring specific protein biomarkers in noninfectious samples may enable safe, point-of-care diagnosis of diseases in resource-limited settings.^11–13^ However, accurate measurement of disease biomarkers in biofluids often requires quantifying proteins or other analytes at ultralow concentrations, which remains a significant challenge.^14^ For pathogens such as *M.tb*, several types of bacterial proteins are secreted into the lungs and released in sputum. However, once these proteins enter the peripheral blood and are excreted in the urine, they become highly diluted and are often undetectable by conventional protein quantification methods.^15–17^ In the case of cancers and neurological diseases, several proteins are highly expressed in diseased tissues in the early stages of disease. However, these proteins become diluted in peripheral blood and remain undetectable in clinical samples by most conventional methods for protein quantification.^16,18,19^ Therefore, alternative diagnostic workflows must be both accessible to patients in resource-limited settings and sufficiently sensitive to detect these low-abundance biomarkers.

Enzyme-linked immunosorbent assay (ELISA), the most widely used method for measuring proteins in biofluids, has a limit of detection (LOD) in the picomolar range (pM or <10^–12^ M), which is not sufficiently sensitive for measuring many disease biomarkers.^14^ To improve upon the LOD of ELISA, several ultrasensitive protein detection technologies have emerged, including single molecule arrays (Simoa),^20^ droplet digital ELISA (ddELISA),^21^ molecular on-bead signal amplification for individual counting (MOSAIC)^22–24^ and nucleic acid-linked immunosandwich assay (NULISA).^25^ Some of these technologies quantify protein biomarkers at ultralow concentrations using a single molecule detection approach in which individual protein molecules are first isolated from the sample into discrete compartments (*e.g*., microwell arrays or droplets) that contain either one or zero molecules. Next, the analytical signal (*e.g*., fluorescence) is measured from each compartment and compared with the background signal to enable digital counting. If there is sufficient signal above background in a given compartment, then it is labeled “*ON*” while others are categorized as “*OFF*”. Using Poisson statistics and the ratio of “*ON*” versus “*OFF*” compartments, it is possible to precisely determine the concentrations of proteins. Single molecule detection has been used to develop a variety of different workflows, with multiple approaches utilizing antibody-coated paramagnetic beads to capture and isolate single molecules. Biotin conjugated detector antibodies are typically added to beads to form sandwich immunocomplexes with captured target molecules. For Simoa and ddELISA, signal amplification is accomplished by labeling immunocomplexes on the beads with an enzyme to catalyze the turnover of many fluorogenic substrate molecules into fluorescent product molecules, localizing them in sealed microwells or droplet compartments. For MOSAIC, sandwich immunocomplexes on beads are labeled with a nondiffusible fluorophore conjugated concatemer using rolling circle amplification (RCA), eliminating the need for compartmentalization and bead isolation. Each of these technologies achieve at least a 1000-fold improvement in analytical sensitivity for protein biomarkers (detection limits at subfemtomolar, *i.e*., <10^–15^ M) when compared to conventional immunoassay methods (ELISA). These methods have been successfully used for protein based disease detection including cancers,^18,26^ neurodegenerative diseases,^27^ and infectious diseases.^28–31^ Single-molecule assays hold great promise for diagnosing these diseases; however, they depend on advanced instrumentation, robust infrastructure, reliable power, and consistent supply chains, which are typically only available at laboratories in high-income countries.^32,33^

Adapting single molecule detection workflows for point-of-care (POC) use in low- and middle-income countries (LMICs) continues to be a significant challenge. At the POC, particularly in LMICs, resource limitations make it difficult and costly to implement temperature-controlled incubations, cold storage for reagents, and sensitive imaging equipment.^34–37^ According to the World Health Organization, an ideal assay for the POC should have a simple workflow, multiplexing capabilities, robust temperature-stable reagents, high sensitivity and selectivity, and low cost.^38^ Several studies have reported single molecule assays that utilize equipment designed for the POC, including portable microscopes and digital microfluidics platforms.^39–41^ These workflows streamline the liquid handling and imaging steps. However, many assays still rely on enzyme amplification (*e.g*., RCA) to label beads and require temperature-controlled incubations and cold storage of reagents, making them poorly suited for widespread deployment. These challenges highlight the need for enzyme-free methods, which eliminate cold-chain dependence and complex incubation steps, making them more practical for deployment in diverse settings.^42^ One of the most promising approaches for enzyme-free signal amplification of biomolecules is the hybridization chain reaction (HCR).^43^ Several studies have reported the utilization of HCR with fixed tissue imaging for the detection of nucleic acids,^44–46^ proteins,^47–49^ small molecules,^43,50^ and proximity-based interactions.^51,52^ An enzyme-free signal amplification approach for single- molecule workflows that is designed to be utilized at the POC would enable many diagnostic applications.

Here, we report a new assay format with a simple workflow, including a rapid labeling approach that does not require cold storage or incubation at elevated temperatures, thereby creating an ultrasensitive protein assay that is designed for use in remote resource-limited clinics. Building on the foundation of Simoa and MOSAIC, we demonstrate that HCR-assembled, dye-labeled oligonucleotide signal amplifiers are suitable labeling reagents for the ultrasensitive detection of different cytokines. We demonstrate this new detection strategy achieves sensitivities that match Simoa, the gold standard method for digital ELISA, with low cross-reactivity. We demonstrate that amplifier reagents are stable at 21 °C and in cold storage for up to six months with no loss in performance. Additionally, we validated this method for detecting proteins in healthy human plasma. Finally, we used dye-encoded beads to demonstrate that a rapid labeling approach can be used for the multiplexed detection of multiple biomarkers simultaneously.

## Results and Discussion

### Development of the Enzyme-Free Single Molecule Detection Platform

At the POC and in LMICs, detection of disease biomarkers in clinically actionable timeframes requires rapid, sensitive assays with simple workflows. To create a simple and accessible ultrasensitive assay, we designed the efMOSAIC workflow to be compatible with instrumentation that is widely available in many settings, *e.g*., 96-well plate washers and flow cytometers.

An overview of the enzyme-free signal amplification reagent and the efMOSAIC assay workflow is presented in Figure 1. efMOSAIC uses the same protein capture workflow and gating strategy as MOSAIC, Figures S1 and S2.^22^ First, antibody coated paramagnetic beads are incubated with samples to capture individual protein molecules. Beads are subsequently incubated with biotin-conjugated detector antibodies to obtain beads with individual sandwich immunocomplexes. In MOSAIC, these immunocomplexes are labeled with streptavidin that is conjugated to a preannealed RCA primer-template pair followed by *in situ* signal amplification by RCA. In efMOSAIC, immunocomplexes are labeled with streptavidin that has been preconjugated to highly fluorescent signal amplifiers, thereby eliminating the need for *in situ* amplification. The streptavidin-tagged signal amplifier (SSA) reagent permits quantitative analysis of complete immunocomplexes by flow cytometry and is generated using isothermal enzyme-free HCR.^43^ To generate the signal amplifier, a pair of metastable, dye-labeled DNA hairpin molecules (H1 and H2) undergo successive hybridization events in the presence of an initiator oligonucleotide (I) that is conjugated to streptavidin, forming a large, nicked double-stranded DNA molecule, Figure S3. The resulting product of this reaction is a dye-labeled nicked DNA signal amplifier containing a terminal streptavidin.

**Figure 1:**
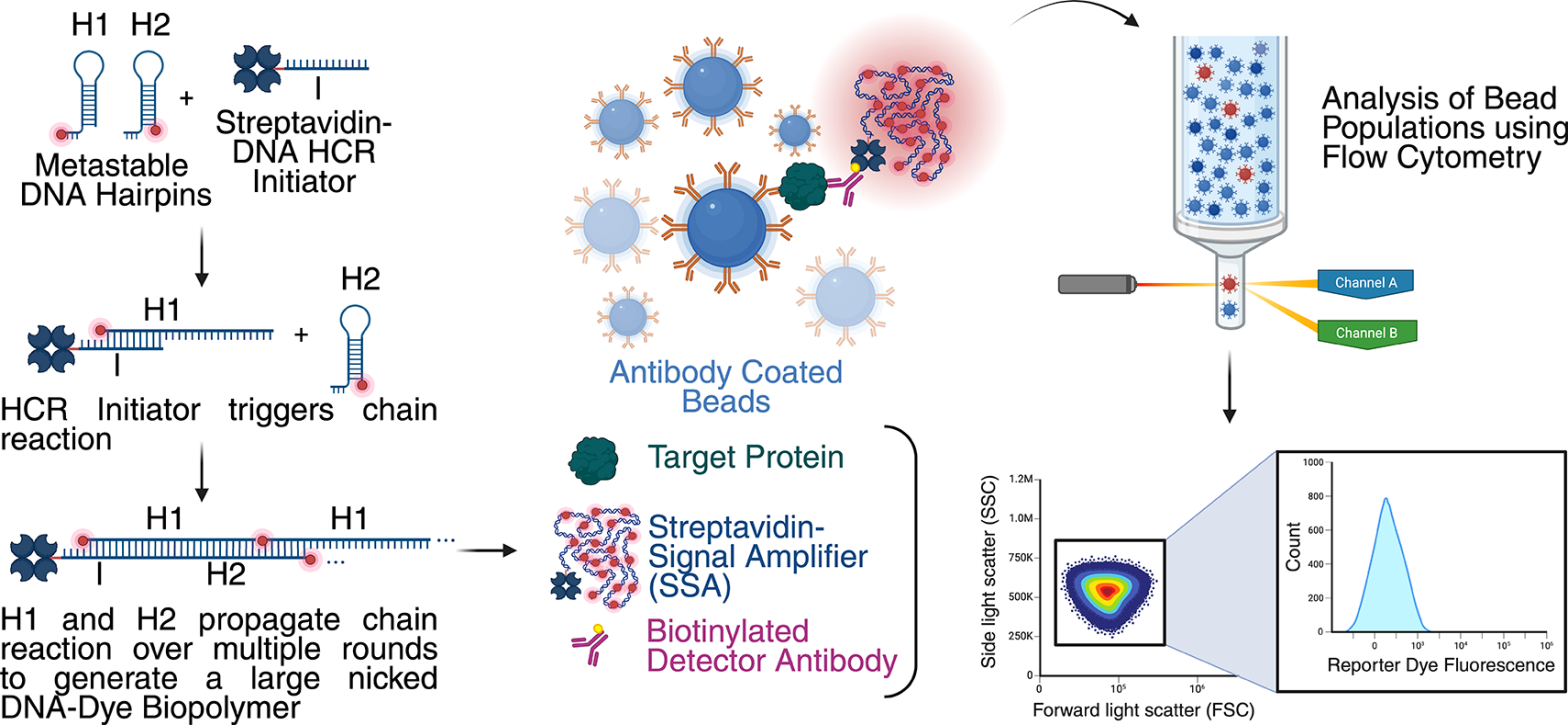
Overview of the enzyme-free signal amplification reagent preparation and the efMOSAIC assay workflow. Streptavidin-tagged HCR initiator oligonucleotides were incubated with dye-labeled hairpin oligonucleotides (H1 and H2) to create a fluorescent signal amplifier. Separately, magnetic beads coated with capture antibodies were first incubated with samples containing target proteins of interest. These beads were isolated, washed, and incubated with biotinylated detector antibodies for immunocomplex formation. The streptavidin signal amplifier was then added to label the biotinylated detector antibodies. The resulting beads were analyzed on a flow cytometer, and the distribution profile of the bead populations was used to determine the concentrations of biomarkers in the samples.

Many of the parameters developed for HCR, including monomer sequence, size, amplification conditions, wash buffers, and reaction temperature, have been optimized for imaging fixed tissue samples.^53–55^ However, bead-based single molecule detection is not constrained by these design considerations and many parameters were reoptimized (data not shown). Suitable dye labels and 96-well plates (*e.g*., nonbinding or medium binding coatings), and sources of background signal from nonspecific binding of signal amplifiers were investigated, Figures S4–S6. In efMOSAIC, all assay steps, including labeling immunocomplexes, are performed at room temperature. The labeling steps are accomplished with 15–30 min of incubation and an accelerated version of the assay can process an entire 96-well plate in 90 min, Figure S7 and Table S1.

### Signal Analysis Using Flow Cytometry

The MOSAIC method utilizes single- molecule counting with flow cytometry to quantify proteins at ultralow concentrations and the fluorescence intensity of the signal amplifiers localized on beads is used to generate a population distribution that is processed using ratiometric analysis for digital detection of proteins. Beads that have sufficient signal above background from fluorescent labels are categorized as “*ON*”, while all other beads are “*OFF*”. In an ideal labeling scenario, there is sufficient signal above background for *“ON”* beads, such that these two subpopulations of beads are well separated and thresholding at a given fluorescence intensity can enable labeling of beads as either *“ON”* or *“OFF*”. Using the ratio of “*ON*” versus “*OFF*” beads and by applying Poisson statistics, it is possible to determine the concentration of proteins with high precision. One of the motivations for using *in situ* enzyme amplification with MOSAIC was to obtain high signal-to-background ratios (SBR) for the digital detection of proteins. However, when there is high fluorescence background on beads, or insufficient signal from fluorescent labels, the population of *“ON”* and *“OFF*” beads are not well separated and *“ON”* beads may be incorrectly counted as *“OFF”* beads, or *vice versa*. Background signal can arise from many sources, including nonspecific binding of fluorescently labeled amplifiers to bead surfaces. To eliminate background in efMOSAIC, stringent washes are used to destabilize any nonspecifically bound amplifiers, however, these conditions can also destabilize the signal amplifiers generated by HCR.

In efMOSAIC, bead-intensity fluorescence is evaluated by the median of the population, rather than digitization. The signal amplifiers used in efMOSAIC workflows have lower fluorescence signal than labels generated with RCA. Due to the lower signal of efMOSAIC amplifiers, single molecule counting yields distributions that have significant overlap between subpopulations of *“ON”* and *“OFF”* beads, evident in distribution of single bead events acquired by flow cytometry (Figure 2(a)). If efMOSAIC were to be analyzed with digitization as in MOSAIC, many *“ON”* beads would be miscategorized as *“OFF”* resulting in poor detection limits. To overcome the challenge of overlapping subpopulation of beads, we explored an alternative method to quantify proteins. Instead of the ratiometric approach used with fluorescence thresholding, we analyzed the median fluorescence intensity of all beads in the entire population distribution (average fluorescence on beads, AFB). The median in this measurement is the middle value of the population distribution, where all bead events are arranged sequentially by fluorescence intensity. As the population of *“ON”* beads increases, the median fluorescence intensity for the distribution increases. When compared to *thresholding “ON” beads*, the AFB captures changes to the entire distribution and does not require well separated subpopulations of *“ON”* and *“OFF”* beads. In efMOSAIC, the fluorescence signal on beads increases in a stoichiometric manner with analyte (and labels). Unlike ensemble measurements (*e.g*., fluorescence intensity in a plate well), AFB requires single molecule analysis of beads to obtain the fluorescence intensity on beads. This analysis approach is similar to the “analog” AEB value used in Simoa but without normalization to the fluorescence intensity of individual labels.^56^ To validate this approach, comparative analysis between the two workflows, *thresholding “ON” beads* and *population distribution analysis*, was done by processing data obtained by efMOSAIC, Figures 2(b) and (c). Calibration curves were generated using both analytical workflows and the analytical sensitivity of the *population distribution analysis* approach was found to be at least an order of magnitude better (LOD = 7 fg/mL versus 90 fg/mL) than the conventional *thresholding “ON” beads* approach. Furthermore, as the amount of analyte increases and each bead has more than one label (*f*_*ON*_ greater than 99.9%), the fluorescence intensity on beads increases stoichiometrically until saturation. The resulting AFB values extend beyond the digital limits of *thresholding “ON” beads* enabling a larger concentration range than digital analysis. Therefore, single-molecule counting and population distribution analysis by AFB can overcome the lower signal from enzyme-free labels, enabling protein detection at ultralow concentrations by flow cytometry. Accessible instrumentation and simple assay workflows can greatly accelerate the adoption of new diagnostic approaches in LMICs. For example, compact cytometers and cell counters have been deployed in LMICs to help manage HIV-positive patients at the POC.^57^ Additionally, compact versions of flow cytometers are being developed for single -molecule detection workflows.^58^ Therefore, adapting the efMOSAIC workflow for use with compact, accessible flow cytometers would enable many diagnostics applications at the point of need.

**Figure 2:**
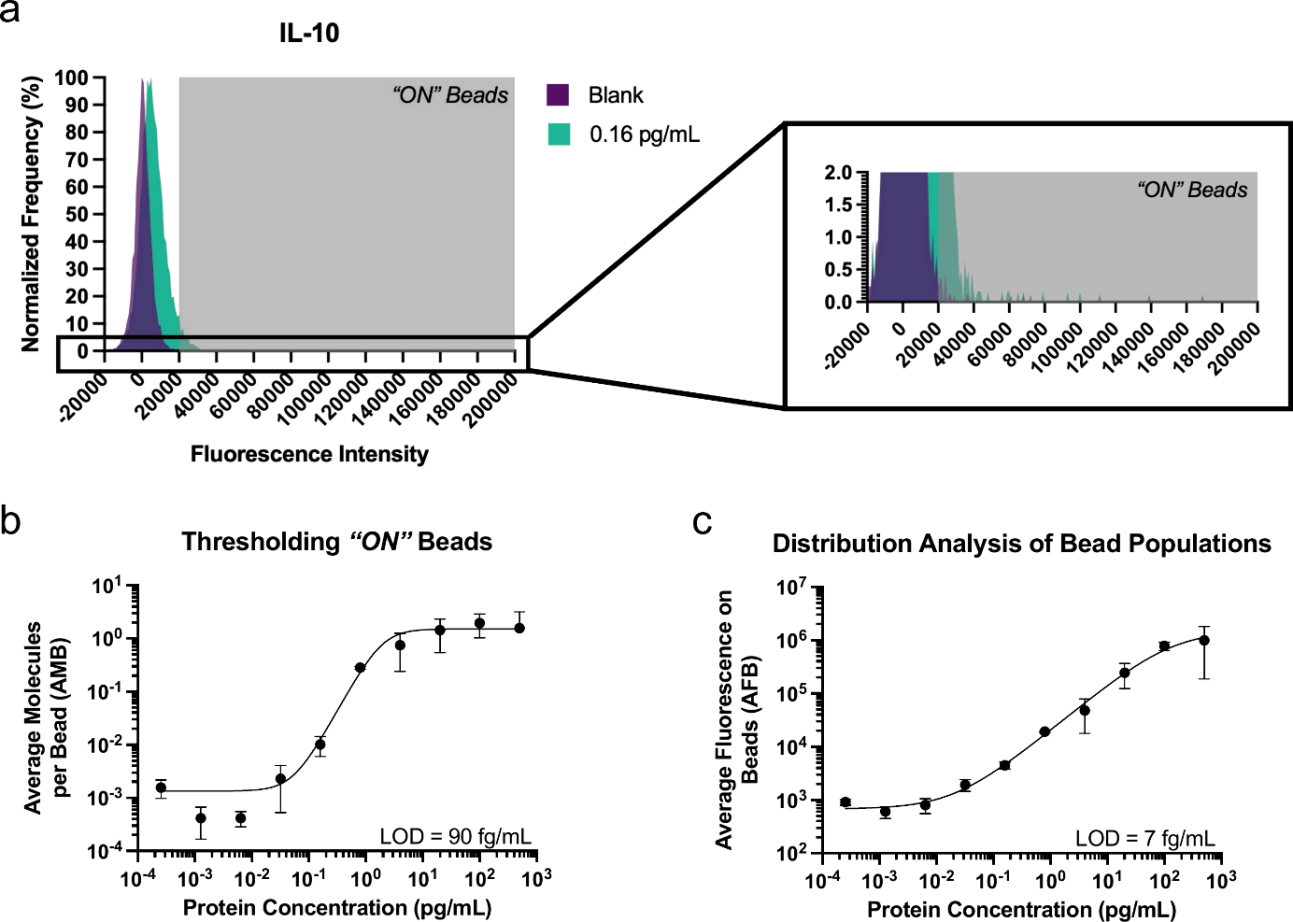
Comparison of sensitivities between thresholding*“ON”* beads and the distribution analysis method for data analysis of efMOSAIC assays. (a) Normalized population distributions from single bead events were acquired for two different calibrators of IL-10 (Blank, and 0.16 pg/mL of IL-10 protein standard in buffer). A threshold (gray box), to discriminate *“ON”* beads from the *“OFF”* beads, was marked as three standard deviations above the mean fluorescence intensity of the Blank population. (Inset) magnified region of the frequency distribution plot showing the population of beads captured in the threshold. (b) The threshold method was used to determine the fraction of *“ON”* beads for a series of calibrators for the IL-10 assay. The average molecules per bead (AMB) value was derived from the *“ON”* bead population and plotted as a function of protein concentration. (c) The average fluorescence on beads (AFB) value was acquired by determining the median fluorescence intensity of the population of single bead events for each calibrator. For both calibration curves, each standard was determined as the average of three independent samples (six for the blank) and curves were fitted using the four-parameter logistic (4PL) regression bead population and plotted as a function of protein concentration.

### Ultrasensitive Detection of Proteins Using efMOSAIC

To evaluate the performance of efMOSAIC, we first developed singleplex assays for seven cytokines (IL-8, IL-10, IL-6, IL-12p70, IL-1β, TNF-α, and IFN-γ). This class of proteins is well-studied and known to be associated with various inflammatory disorders,^59^ such as cancer,^24,60^ and infectious diseases.^61,62^ Cytokine measurements in clinical biofluids have also been performed using other single-molecule detection approaches (*e.g*., Simoa, MOSAIC), facilitating direct benchmarking of performance across the different techniques.^22,63^ We conducted a parallel set of measurements on the Simoa and efMOSAIC platforms using the same preparation of reagents, Figure 3(a). Each set of calibration curves was run on three different days and averages of the performance metrics are summarized in Tables 1, S2–S4, and Figures S8–S21. efMOSAIC assays for IL-10 and IL-6 were found to have similar LODs to Simoa, while other cytokines, such as IL-8, IL-12p70, and IL-1β had marginally lower (<5×) performance in the efMOSAIC format. In contrast, TNF-α, and IFN-γ efMOSAIC assays had 5× and 30× better LOD than Simoa for the same assays. We further analyzed these trends with comparative studies of the SBR for each set of assays with Simoa and efMOSAIC (Figure S22). In general, IL-6 had a similar SBR for both methods, while Simoa had better SBR over the calibration ranges for IL-10, IL-8, IL-12p70, and IL-1β. TNF-α, and IFN-γ, had better SBR for efMOSAIC, which supports the trends observed in Table 1. When we compare the day-to-day variations in LOD for the different assays (Table S4), we find that efMOSAIC is more variable than Simoa for three of the assays (IL-8, IL-10, IL-12p70) and less variable for four of the assays (IL-6, TNF-α, IL-1β, IFN-γ). Additionally, we do not observe any significant changes in the shape of the calibration curves performed on different days indicating that efMOSAIC is not more variable in performance than Simoa (Figures S8–S21). Next, specificities of the efMOSAIC assays were investigated by comparing the SBR of samples with target and nontarget proteins, summarized as a heat map (log scale) in Figure 3(b). Significant interference from antibody cross-reactivity, nonspecific binding of signal amplifiers, or other cross-reacting molecules would result in nonspecific signals. No significant increase in the SBR was observed for any assay, suggesting good specificity and low cross-reactivity for the singleplex variants of the efMOSAIC assays. Compatibility of the efMOSAIC workflow with additional flow cytometers (Cytoflex S and LX) was also investigated using calibration curves for the IL-10 assay (Figure S23 and Table S5). One of the flow cytometers we investigated, the Cytoflex S, is a small lightweight benchtop system with robust optics that is well suited for use in resource-limited settings. IL-10 assays were performed without modifications from the standard protocol and readout obtained from the two additional flow cytometers indicated no loss in assay performance. The analytical performance of efMOSAIC relative to Simoa varies depending on the target protein. Although efMOSAIC may exhibit marginally lower sensitivity for certain proteins, its advantages in assay workflow simplicity and long-term reagent stability make it well suited for POC applications.

**Figure 3:**
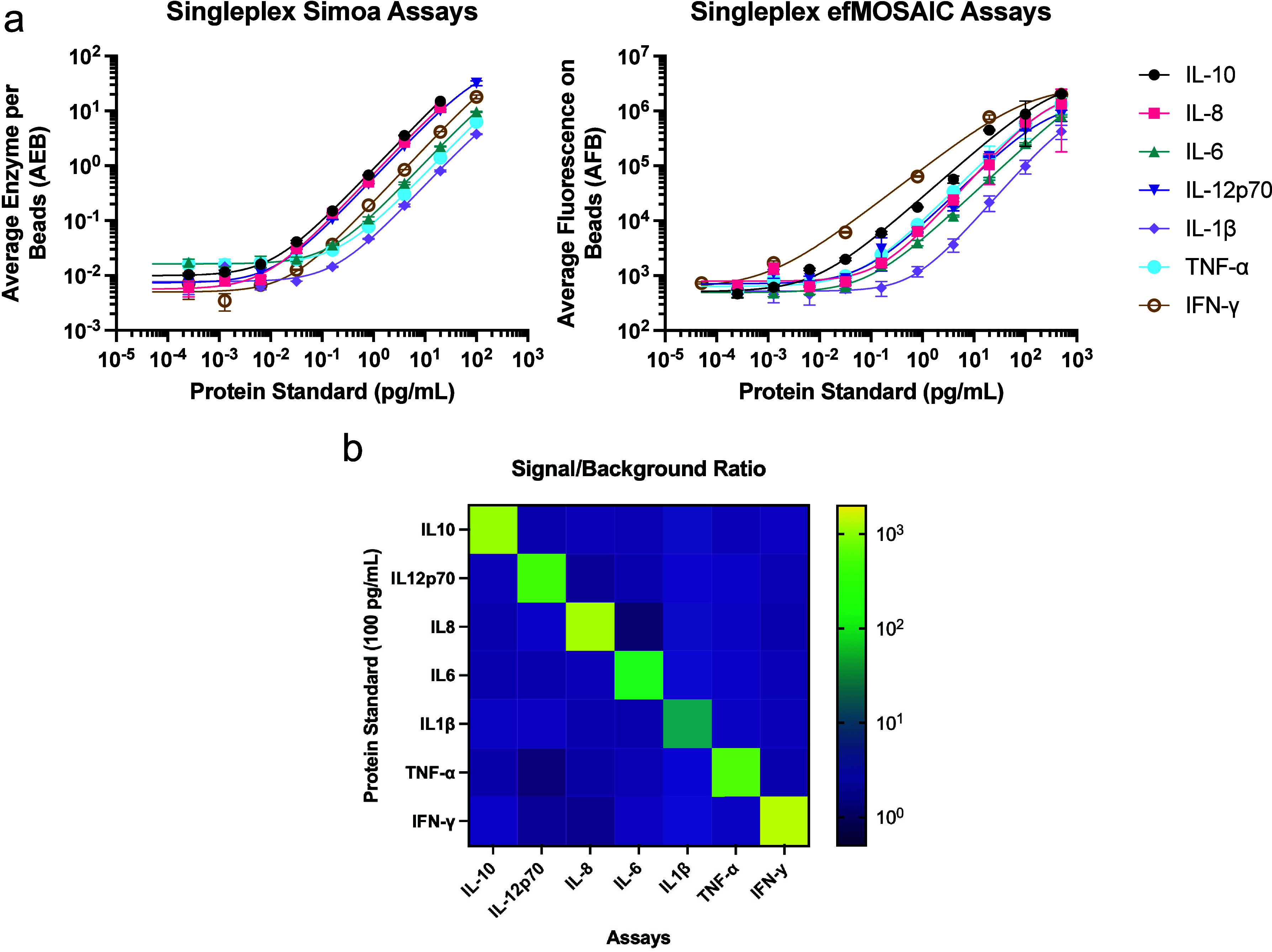
Comparison of performance between the Simoa and efMOSAIC methods. (a) Calibration curves for seven different inflammatory protein biomarkers (including IL-10, IL-8, IL-6, IL-12p70, IL-1β, TNF-α, IFN-γ) were obtained in the Singleplex format using Simoa and efMOSAIC. (b) The signal-to-background ratios (SBR) of efMOSAIC assays with 100 pg/mL of target and nontarget protein standards were measured. Here, elevated SBRs in nontarget samples would indicate assay cross reactivity. All calibration curves were fitted using the four-parameter logistic (4PL) regression and error bars were standard deviations from three replicates.

**Table 1:** Limit of Detection (LOD) and Lower Limit of Quantification (LLOQ) for efMOSAIC, and the Corresponding Simoa Assays^a^

	Limit of Detection (fg/mL)		Limit of Quantification (fg/mL)	
Analyte	efMOSAIC	Simoa	efMOSAIC	Simoa
IL-8	20 ± 9	3 ± 2	120 ± 46	10 ± 5
IL-10	5 ± 4	2 ± 1	28 ± 17	8 ± 3
IL-6	40 ± 17	30 ± 34	206 ± 129	98 ± 132
IL-12p70	20 ± 10	4 ± 5	104 ± 22	12 ± 19
IL-1β	364 ± 55	73 ± 71	1244 ± 613	262 ± 212
TNF-α	22 ± 9	114 ± 46	70 ± 35	349 ± 144
IFN-γ	0.14 ± 0.04	4 ± 3	2 ± 1	24 ± 6

a LOD and LLOQ are measured as three and ten SD above noise, respectively. Average of three calibration curves performed on three different days.

### Long-Term Stability of SSA Reagent

By eliminating enzyme-based amplification, efMOSAIC relies on stable, preformed signal amplifiers rather than in situ enzymatic reactions. Importantly for suitability in POC settings, the SSA reagent is generated in a preamplification step at 21 °C and can be stored in the dark at ambient temperature for several months until it is used to label biotin-linked immunocomplexes. We investigated the SSA reagent stability for long-term storage by evaluating calibration curves for the IL-10 protein with freshly prepared and stored aliquots of the signal amplifier. Measurements of the assay were done biweekly from SSA reagents that were stored at −20, 4, and 21 °C. SSA reagent that was stored in the dark at 21 °C for up to six months had no loss in performance when compared to freshly prepared SSA, Figure 4. Separately, SSA reagents that were stored in the dark at −20 and 4 °C were also stable for up to six months with no loss in performance, Figure S24, Table S6. While the assay requires beads and detector antibodies that are prepared at low temperatures, once they are prepared, these reagents can be kept at ambient temperatures. Furthermore, others have shown that temperature sensitive reagents can be formulated into tablets for improved stability and controlled release of reagents in multistep assays.^64,65^ Unlike enzymatic approaches, efMOSAIC reagents do not require temperature-controlled incubations or cold-chain logistics. Additionally, signal amplifiers show no interference from nonspecific binding or cross-reacting molecules, and most 96-well plates can be processed within 90 min for readout.

**Figure 4:**
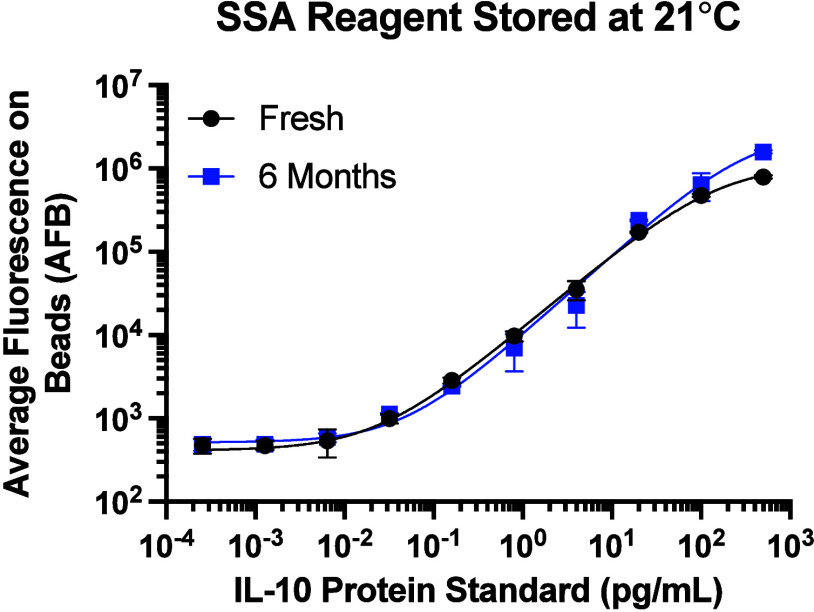
Long-term stability of SSA reagent at 21 °C. The stability of the efMOSAIC SSA reagent was investigated by measuring the signal response of the IL-10 assay using freshly prepared SSA reagent, and a batch that was stored in darkness at 21 °C for six months. All calibration curves were fitted using the four-parameter logistic (4PL) regression and error bars were standard deviations from three replicates.

### Detecting Proteins in Human Plasma Samples

efMOSAIC was validated to quantify endogenous proteins in human plasma by spike and recovery and dilution linearity (parallelism). For dilution linearity studies, proteins for each assay were measured in three different lots of individual human plasma that were diluted between 2× to 32× with sample diluent. A summary of the pooled dilution corrected concentrations for each biomarker is given in the SI (Figure S25, Tables S7–S15). For most assays, the concentrations of measured protein for successive dilutions were within 80–120% of the expected concentration.^66^ Nearly all the proteins quantified by efMOSAIC demonstrated strong linear trends during dilution, suggesting minimal impact on assay performance from matrix effects. Separately, spike and recovery measurements were also done in contrived samples consisting of 8× diluted plasma with one of three different concentrations of recombinant protein standards (low, medium, or high spike). The amount of spiked protein varied between assays to account for differences in the expected endogenous concentration of each protein in plasma. For most proteins, a recovery range of 80–120% was obtained, suggesting minimal impact from the plasma matrix. IFN-γ had the lowest recovery. We believe that bead aggregation may have contributed to bead loss during sample measurements, leading to the lower recovery.

### Multiplexed Detection of Proteins

Multiplexed antigen detection offers advantages over single-analyte assays by capturing a broader range of biological information, reducing diagnostic uncertainty, and enabling more robust performance across diverse patient populations.^74–76^ This approach is particularly useful when no single biomarker can reliably distinguish disease states.^77^ For example, in inflammation, individual antigens often lack sufficient sensitivity or specificity on their own, but panels of proteins have shown improved diagnostic accuracy.^73^ Thus, multiplexed detection strategies hold significant promise for enhancing clinical utility, especially in resource-limited settings where maximizing information from limited sample volumes is critical. Future diagnostics tests for other complex multifactorial diseases such as cancers and neurodegenerative diseases would also require panels of multiple biomarkers to accurately diagnose patients.^78^ In all these clinical scenarios, measuring multiple biomarkers from a single sample with the efMOSAIC platform would greatly improve accessibility of testing to patients in LMICs.

In Simoa and MOSAIC, bead color and size have been used to encode a multiplex panel of eight different targets.^22,79^ In the present study, we explore the performance of the SSA labeling reagent, so we limited our proof-of-concept study to a duplex assay to measure IL-10 and TNF-α. To simultaneously measure multiple proteins from the same sample, we used dye-encoded beads (λ_max_^*EM*^ = 488 and 750 nm) conjugated with antigen-specific capture antibodies, Figure 5(a). Bead color was used to identify the different assays during gating, and quantification was done in a similar manner to singleplex assays. When both proteins were present in the calibration curve, efMOSAIC assays had a slightly higher LOD (<3–5×) and largely the same dynamic range compared to the singleplex efMOSAIC assays and multiplexed Simoa assays, Figures 5(b, c), S26–S31, and Tables 2, S16, and S17.

**Table 2:** Summary of Multiplexed Assays and Protein Dropout for efMOSAIC and Simoa^a^

		Limit of Detection (fg/mL)		Lower Limit of Quantification (fg/mL)	
Assay Format	Bead Channel	efMOSAIC	Simoa	efMOSAIC	Simoa
Both Proteins	IL-10	24 ± 17	5 ± 4	84 ± 61	15 ± 13
	TNF-α	145 ± 86	53 ± 71	534 ± 356	192 ± 249
TNF-α only	**IL-10**	**Low Crosstalk**	**Low Crosstalk**	**Low Crosstalk**	**Low Crosstalk**
	TNF-α	66 ± 62	299 ± 192	234 ± 133	1040 ± 531
IL-10 only	IL-10	7 ± 2	3 ± 2	33 ± 8	10 ± 4
	**TNF-α**	**(0.92 ± 0.54) × 10** ^**4**^	105 ± 95	**(3.50 ± 2.50) × 10** ^**4**^	380 ± 365
		**Low Crosstalk**	**High Crosstalk**	**Low Crosstalk**	**High Crosstalk**

a LOD and LLOQ are measured as three and ten SD above noise, respectively. Average of three calibration curves performed on three different days.

**Figure 5:**
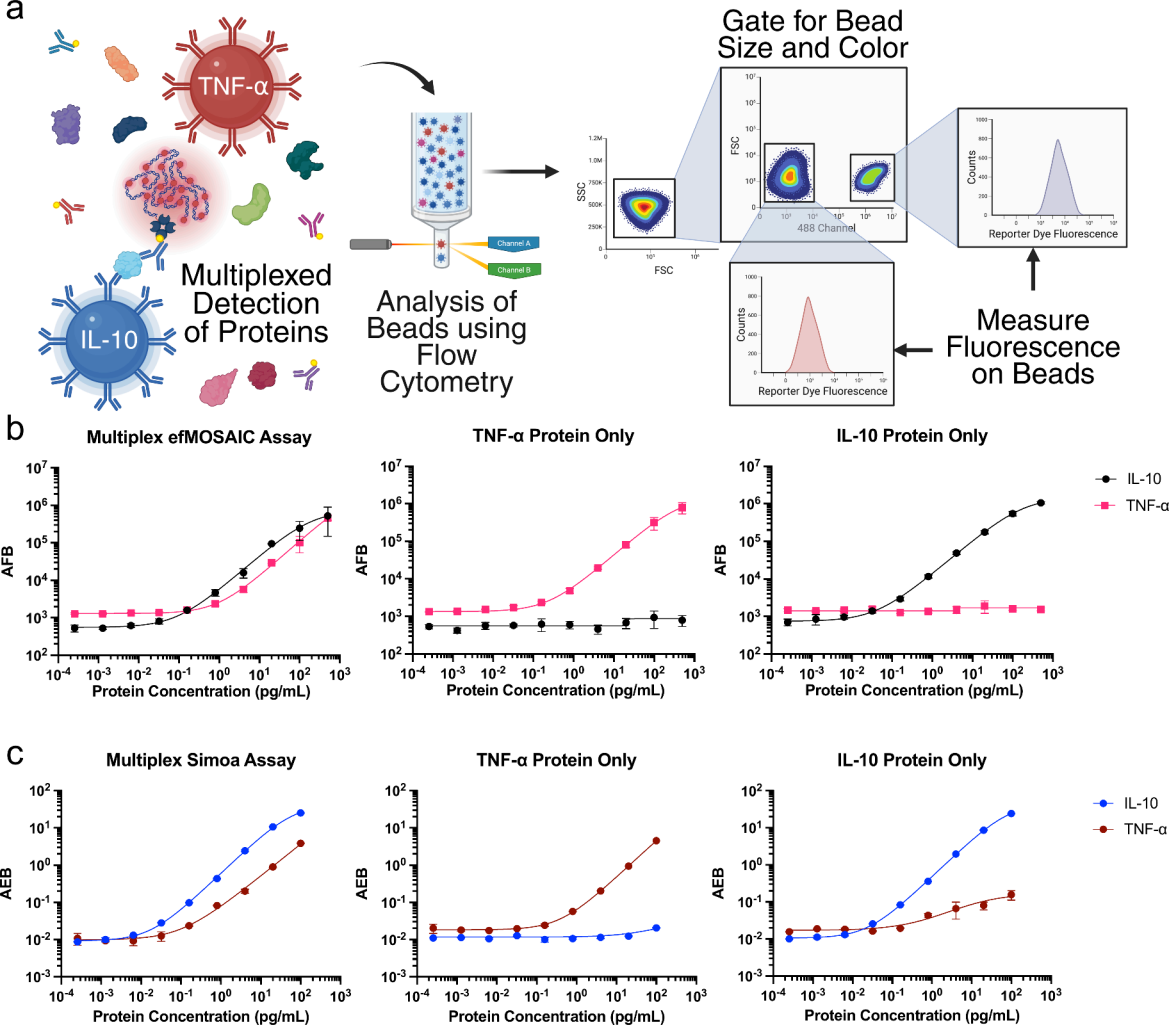
Comparison of sensitivity and cross-reactivity between duplex Simoa and efMOSAIC assays. (a) Overview of the multiplexing workflow and gating strategy for dye-encoded paramagnetic beads (***λ***_***Max***_^***EM***^ = 488 and 750 nm) that were conjugated with different capture antibodies (IL-10 and TNF-α, respectively). Calibration curves, and protein dropout curves (TNF-α or IL-10 protein only) were obtained for the (b) efMOSAIC assays, and the complementary (c) Simoa assays. Representative curves were fitted using the four-parameter logistic (4PL) regression and error bars are standard deviations from three replicates for samples and blanks.

Protein dropout experiments were also performed to quantify sources of nonspecific binding and cross-reactivity in multiplexed efMOSAIC assays. One of the technical challenges of simultaneously measuring multiple proteins is reducing unexpected off-target binding events. For example, cross-reactivity of antibodies with off-target proteins can lead to false positive signals and poor measurement accuracy in ultrasensitive detection assays. In Simoa, these effects have been addressed by sequential protein capture of cross-reactive assays.^80^ To investigate the extent of cross-reactivity in multiplexed efMOSAIC, we performed protein dropout experiments where only one target protein (TNF-α or IL-10 protein only) was present in calibrators, Figures 5(b, c), S26–S31, and Tables 2, S16, and S17. In multiplexed Simoa, significant cross-reactivity was observed between color channels for IL-10 and TNF-α when only IL-10 protein was present in calibrators. Interestingly, cross-reactivity was not observed between the color channels in multiplexed efMOSAIC. Additionally, these trends in multiplexed Simoa and efMOSAIC were observed reproducibly when protein dropout experiments were repeated across three sets of measurements on three different days. One possibility for the lower cross-reactivity observed in multiplexed efMOSAIC may be the stringent wash conditions, which contribute to reducing nonspecific binding of amplifiers to beads. However, these observations require further investigation with other assays, and with larger multiplexed panels.

Previous reports have demonstrated that autofluorescence of magnetic beads and signal crosstalk between dye-encoding labels and reporter channels can impact assay performance and that photobleaching magnetic beads can improve the SBR and sensitivity of bead-based assays.^81,82^ In this study, we investigated the effect of photobleaching the capture beads on the background signal and SBR in efMOSAIC. When we compare the assay performance of 488 and 750 dye-encoded magnetic beads that are conjugated with the same type of antibodies, we observe a higher SBR for the 488 dye-encoded beads (Figures S32 and S33). However, after photobleaching the 750 dyed beads, the background of the 750 beads was comparable to that of the 488 beads (Figure S33), and the SBR was similar. Photobleaching 750 dyed beads resulted in a 79% reduction in background signal and an ∼3-fold improvement in the SBR compared to nonbleached 750 dyed beads, (Figures S32 and S33). This technique is applicable to any assay developed on the efMOSAIC platform, offering users an additional method to mitigate background noise and improve the assay performance.

## Conclusions

There is a growing need for accessible diagnostic technologies capable of ultrasensitive biomarker quantification from minimally invasive biofluids.^42^ Ultrasensitive protein detection assays have complex assay workflows that require advanced instrumentation, temperature-controlled incubations, and cold storage of reagents.^32^ These requirements limit their applicability in low-resource and POC settings. To address these challenges, we developed efMOSAIC, which labels single molecules under ambient conditions using enzyme-free signal amplifiers generated by HCR. This design eliminates enzymatic amplification and cold-chain logistics, while enabling complete assay workflow to be performed at 21 °C with a 90 min turnaround time on standard flow cytometry platforms.

As a proof-of-concept, we tested this labeling strategy for a series of singleplex assays for detecting several different cytokines. Two efMOSAIC assays (TNF-α and IFN-γ) had superior LODs, three assays (IL-8, IL-12p70, and IL-1β) had marginally higher LODs, and the remaining assays (IL-6 and IL-10) had similar LODs to the gold-standard Simoa assays. Performance variations for assays were validated with multiple replicates over several days and similar trends in the SBR were observed for both efMOSAIC and Simoa. Additionally, we found that signal amplifiers were stable when stored in the dark at 21 °C for up to 6 months with no loss in performance. Cold storage of assay reagents greatly increases infrastructure requirements in LMICs. Transporting materials to resource-limited settings is more challenging when reagents need to be stored at 4, −20 or −80 °C because they require continuous energy input and any delays or disruptions in transport can lead to loss of reagents and assay performance.^83^ The increased cost of transport and storage adds an additional barrier to the end-user, limiting wider implementation of these technologies. While there are reports of lyophilized enzymes and reagents being used to develop POC-friendly formulations of reagents, temperature-controlled incubations are still required for signal amplification, which is not ideal for simple workflows.^83^

We investigated the performance of efMOSAIC for the ultrasensitive detection of endogenous and spiked proteins in human plasma samples. We observed minimal differences between the measured and expected concentrations of most cytokines, with the exception of IFN-γ which had lower yields for all spikes. Our findings suggest that efMOSAIC is a suitable platform for measuring proteins from plasma samples over a wide range of dilutions. Having demonstrated the clinical utility of efMOSAIC for measuring cytokines, we believe that there is potential to expand the workflow to measure other biomarkers of disease. Furthermore, the efMOSAIC workflow should be compatible with other biofluids that have been investigated with MOSAIC (*e.g*., serum and saliva) because the sample processing steps are similar across the two platforms.

It is often useful to measure multiple proteins simultaneously because a combination of biomarkers often has better predictive value for a diagnostics test than single biomarker measurements. Therefore, we developed a proof-of-concept duplex assay to investigate the multiplexing capabilities of efMOSAIC. Most notable, in protein dropout experiments for this duplex assay, Simoa had high cross-reactivity which can lead to false positive measurements of TNF-α. efMOSAIC had minimal cross-reactivity in the complementary set of assays which may be due to the additional number of wash steps in the assay workflow. This duplex assay demonstrated the feasibility of the dye-labeling strategy for multiplexing with efMOSAIC, and we anticipate expanding to higher levels of multiplexing will expand clinical utility of this method. Additionally, we validated photobleaching as a suitable strategy for improving the SBR of dye-encoded beads when there is signal crosstalk between dye label and reporter fluorescence channels.

Despite eliminating enzymatic amplification from the assay workflow, additional work is still needed to improve accessibility of efMOSAIC to LMICs. For example, the work presented herein relies on a plate washer and flow cytometer to process and analyze samples. Transitioning efMOSAIC to LMICs will require further validation with integrated lab-on-chip devices, portable microscopes, and simpler flow cytometers that are more suitable for resource-limited settings. These simpler instrumentations may have additional impact on the analytical performance of efMOSAIC workflows. Early testing with three different flow cytometers has not shown any loss in performance or instrument-specific variations. However, a flow cytometer with lower power lasers and less sensitive detectors may not generate sufficient signal for this assay. Therefore, it will be important to validate the performance of the efMOSAIC workflow with additional flow cytometers that are already deployed in LMICs. While we investigated the stability of the labeling reagent, further investigation is required to test the limits of stability for antibody coated beads, detector antibodies, and protein calibrators. Additionally, many clinically relevant biomarkers are present at concentrations below the current detection limits of efMOSAIC. The development of new, high-affinity antibodies to capture and label these biomarkers will be essential for further improving assay performance and diagnostic utility. Brighter enzyme-free labels that can enable digital counting may also improve the performance of single molecule detection assays.

In summary, we have developed an ultrasensitive enzyme-free approach for single molecules detection of proteins. This method has a simple assay workflow and uses bright signal amplifiers to label beads at ambient conditions without enzymes. We demonstrate that assays can be done in human plasma samples and dye-encoded beads can be used for the multiplexed detection of two proteins simultaneously with minimal cross-reactivity. We expect this assay format to be applicable to many different diagnostic applications that are not presently accessible in resource-limited settings.

## Experimental Methods

### Materials

A detailed list of materials and reagents used in this report are listed in the Supporting Information. Antibodies were from Biolegend, R&D Systems, Abcam, or BD Bioscience unless stated otherwise. Synthetic oligonucleotides were from Integrated DNA Technologies.

### Instrumentation

Flow cytometry measurements were acquired with the NovoCyte Flow Cytometer, Model: 3000RYB equipped with the autosampler module NovoSampler Pro, Model: NS200 (Agilent Technologies, San Diego, CA). Additional measurements were also acquired with the Beckman Coulter Cytoflex S V4-B2-Y4-R3 flow cytometer, and the Beckman Coulter Cytoflex LX flow cytometer (Beckman Coulter, Indianapolis, IN). Plate washing was done using the BioTek Instruments plate washer, Model: 405TSRVS with DI water, and Quanterix System Wash Buffer 1 (Quanterix Corp). For efMOSAIC, all measurements were done in the 96-well plate format. Simoa assays were done on the HD-X Analyzer (Quanterix Corp., Billerica, MA) which automated all assay steps, image analysis, and average enzyme on bead (AEB) calculations. The same batches of capture beads and biotinylated detector antibodies were used for all efMOSAIC and SIMOA experiments.

### Preparation of Antibody Coated Magnetic Beads

Antibodies were conjugated to capture beads using EDC chemistry. Pure lyophilized antibodies were first reconstituted in Bead Conjugation Buffer (Quanterix) to a concentration of 0.2 mg/mL. If the antibodies were in a storage buffer that contained other additives, then further processing was done to transfer the antibodies into Bead Conjugation Buffer. Briefly, 100 μg of antibodies in buffer was transferred to a 2.0 mL tube and diluted to a volume of 500 μL with Bead Conjugation Buffer. Next, the solution was mixed gently and transferred to an Amicon centrifugal filter unit (MWCO = 50 kDa) and centrifuged at 14000 × g for 5 min. The filtrate was discarded, and antibodies were diluted to 500 μL with Bead Conjugation Buffer for additional centrifugation. This process was repeated a total of three times. Antibodies were collected by inverting the Amicon centrifugal filter unit into a collection tube that was centrifuged at 1000 × g for 2 min. The filter was rinsed with 50 μL of MES buffer (in two aliquots of 25 μL) before it was centrifuged again at 1000 × g for 2 min. The antibody solution was then characterized by nanodrop and diluted to a final concentration of 0.2 mg/mL. For short-term storage, antibodies were kept at 4 °C.

Capture beads were removed from cold storage and placed on a hula mixer for 10 min. An aliquot of 2.7-μm capture beads was transferred to a 2.0 mL tube and placed on a magnetic separator. Storage solution was removed and 300 μL aliquots of Bead Wash Buffer (Quanterix) were used to wash beads three times. Next, the Bead Wash Buffer was removed, and beads were washed two additional times with 300 μL of Bead Conjugation Buffer. Finally, beads were redispersed in 300 μL of MES buffer and activated with EDC. To activate beads, EDC (1 mg) was reconstituted with 100 μL of Bead Conjugation Buffer to a concentration of 52.2 mM and vortexed until completely dissolved. Next, 10 μL of the EDC solution was transferred to beads and the mixture was placed on a hula mixer at 4 °C for 30 min. The reaction mixture was aspirated and 300 μL of the Bead Wash Buffer was added to the beads. Beads were then rinsed with an additional aliquot of Bead Wash Buffer before the addition of the 0.2 mg/mL antibody solution. The resulting mixture of beads and antibodies was vortexed and placed on a hula mixer at 4 °C for 2 h. After incubation, beads were purified from unconjugated antibodies. First, the reaction mixture was placed in a magnetic separator, and the antibody solution was aspirated. Beads were then washed with two 300 μL aliquots of Bead Wash Buffer. Next, buffer was removed and a 300 μL aliquot of Bead Blocking Buffer (Quanterix) was added to the beads. The mixture was vortexed and placed on a hula mixer at 21 °C for 45 min. After incubation, the blocking solution was removed, and beads were washed with two aliquots of 300 μL of Bead Wash Buffer before being resuspended in 300 μL of Bead Diluent (Quanterix). Beads were stored in the dark at 4 °C and analyzed the next day using the Beckman Coulter Z1 Particle Counter.

### Photobleaching Multiplex Beads

Dye-encoded magnetic beads (λ_max_^*EM*^ = 750 nm) conjugated with TNF-α antibodies were diluted to a volume of 2 mL (160 × 10^6^ beads) with Bead Diluent (Quanterix) and transferred to a capped 35 mm cell culture dish. The dish was positioned in the photobleaching apparatus and placed on a benchtop plate shaker (Fisher Scientific). The conjugated beads were photobleached at 4 °C with 300 rpm of mixing for 24 h. The MFI of the beads was measured on the flow cytometer both prior to and following photobleaching, Figure S32.^81,82^

### Preparation of Biotinylated Detector Antibodies

Antibodies were biotinylated when the biotin modification was unavailable. 100 μg of pure lyophilized antibodies were dissolved in 500 μL of Biotinylation Buffer (100 mM PBS at pH 7.4, Quanterix) and mixed at 21 °C for 10 min. Next, the antibody solution was purified from additives with three centrifugal cycles (14000 × g for 5 min per run) of the Amicon centrifugal filter units (MWCO = 50 kDa). The retained residue was collected by centrifugation (1000 × g for 2 min per run) and characterized using the Nanodrop Spectrophotometer. Once quantified, the antibodies were incubated with a 40-fold molar excess of NHS-PEG_4_-Biotin (ThermoFisher Scientific) prepared fresh in ultrapure water. The resulting reaction mixture was incubated at 21 °C for 30 min. After biotinylation, the reaction mixture was transferred to an Amicon filter unit (MWCO = 50 kDa) and purified using three centrifugal cycles (14000 × g for 5 min per run). The retained residue was collected by centrifugation (1000 × g for 2 min per run) and quantified using the Nanodrop Spectrophotometer before it was stored at 4 °C until required.

### Synthesis of Streptavidin-Initiator (S–I) Oligonucleotide Conjugates

Streptavidin (1 mg, 189730; Millipore Sigma) was reconstituted with PBS buffer [1 mg/mL] and mixed at 21 °C for 10 min. Separately, DBCO-PEG_4_-NHS (764019; Millipore Sigma) was dissolved in DMSO [5 mg/mL] and placed on a hula mixer at 21 °C for 10 min. An aliquot of DBCO-PEG_4_-NHS (20-fold molar excess) was then transferred to streptavidin, and the mixture was incubated with mixing at 21 °C for 30 min. The streptavidin-DBCO conjugate was purified from excess DBCO-PEG_4_-NHS with five centrifugal cycles (14000 × g for 20 min per run) using the Amicon centrifugal filter units (MWCO = 3 kDa). Streptavidin-DBCO conjugates were collected in PBS buffer using two centrifugal cycles (1000 × g for 2 min per run). The concentration of streptavidin-DBCO was measured using a nanodrop (setting on the nanodrop was (E 1%) = 32) by diluting the stock solution 20-fold. This measurement is an approximation because DBCO will contribute to some amount of signal at A280 (Streptavidin MW of 53 kDa used in calculations). Separately, a 2-fold molar excess of Azide-modified initiator oligonucleotide was added to a fresh 2 mL centrifuge tube along with an aliquot of 10 × PBS to yield a final solution of initiator in 1 × PBS buffer. This solution was then added to the streptavidin-DBCO and incubated with mixing at 4 °C overnight. The next day, the resulting mixture was diluted (normalized to streptavidin concentration = 30 μM) to yield a solution of streptavidin-initiator in 1 × PBS with 0.1% BSA, and 0.02% sodium azide. Aliquots of the solution were placed for storage at −80 °C.

### Preparation of Streptavidin Signal Amplifier by Hybridization Chain Reaction (HCR)

A standard solution of Streptavidin-Initiator (S–I) conjugates was prepared by diluting the stock solution 100-fold with PBS to a final concentration and volume of 0.3 μM in 100 μL. Separately, frozen stocks solutions of ATTO647N dye-labeled hairpins [100 μM], H1, and H2, were thawed in the dark to room temperature. The signal amplifier reagent was then prepared by diluting the standard solution of S–I conjugates, and hairpins in amplification buffer, and incubating the resulting mixture in the dark at room temperature overnight. The amplification buffer consisted of 5 × SSC + 0.1% Tween20 + 10% w/v Dextran Sulfate +0.1 M MgCl_2_ + 0.02% Sodium Azide and was prepared as detailed in the SI. For a typical batch of reagent, S–I, H1, and H2, were diluted to final concentrations of 300 pM, 0.3 μM, and 0.3 μM, respectively.

### efMOSAIC Assays

Capture beads in a stock solution were dispersed and diluted in Sample Diluent (Quanterix) to a concentration of 2 × 10^6^ beads/mL for singleplex assays. Separately, calibrators were prepared by serial dilution of protein standards in Sample Diluent. Ten μL aliquots of capture beads were added to wells in a 96-well plate before the addition of 100 μL aliquots of calibrators. The plate was sealed and placed on an analog shaker (650 rpm) at room temperature for a 1 h. After incubation, the plate was washed six times using System Wash Buffer 1 (Quanterix). The bead residue was redispersed in 100 μL of detector antibody solution, sealed, and incubated with mixing at 650 rpm at 21 °C for 15 min. Next, the plate was washed six times using System Wash Buffer 1 and the bead residue was redispersed in 50 μL of signal amplifier (SSA) reagent. The plate was sealed and incubated with mixing at 650 rpm at 21 °C for 15 min. Next, the samples were diluted with 200 μL of 1 × SSCT wash buffer and incubated on the plate magnet for 2 min. The buffer solution was removed, and the residue was redispersed with 150 μL of 1 × SSCT wash buffer and incubated for 2 min on a plate magnet. Finally, the solution was removed, and the beads were redispersed in 120 μL of 1 × SSCT wash buffer. Multiplex assays were done with the same workflow but using different amounts of 488 and 750 dye-encoded beads. Measurement was done on the flow cytometer or by drop-casting onto glass slides.

### Simoa Assays

For singleplex assays, a mixture of capture (125,000) and helper beads (375,000) were incubated with a 100 μL of sample/calibrator solution. For multiplex assays, an equal mixture of 488 and 750 capture beads (500,000 total) was used. All assays were done in the 2-step format by incubating beads, detector antibodies, and samples for 35 min to form the sandwich immunocomplex. Concentrated stock solution of streptavidin-β-galactosidase (SβG) was diluted to a final working concentration of 150 pM and incubated with beads for 5 min to label single immunocomplexes. Resorufin-β-d-galactopyranoside (RGP; enzyme substrate), sealing oil, system buffer, wash buffer 1, and wash buffer 2 were also loaded into the HD-X Analyzer according to manufacturer instructions. In general, samples were transferred to a 96-well plate and loaded into the HD-X Analyzer along with plastic bottles containing capture beads, biotinylated detector antibodies, and SβG solution.

### Data Analysis

Flow cytometry data was exported in the FSC 3.0 format using the NovoExpress Software Package Version 1.6.2 (Agilent Technologies Inc., San Diego, CA), and analyzed by FlowJo Software (Becton, Dickson, and Company), as summarized in Figures S2 and S3 of this report. The gating workflow was done for negative control beads and then applied to all other wells. First, bead populations were identified by gating in the forward and side scatter plots. Here, single dye-encoded beads were selected and gated for further analysis such that only beads with signal were analyzed for detectors, *e.g*., beads coated with FITC dye molecules (l_EM_ = 488 nm). Next, detector dye molecules on beads were measured for the dye signal, *e.g*., ATTO647N dye signal (l_EM_ = 647 nm). The Gaussian statistical parameters for the distribution of detector signal on beads was calculated and used to determine the LOD and LLOQ, three and ten standard deviations above the background, respectively. Using the thresholding method, beads with and without fluorescent labels are assigned the digital value of “ON” and “OFF”, respectively. The fraction of “ON” beads (*f*_*ON*_) in each sample is converted into an AMB value using eq 1.

1 $$\mathrm{AMB}=-\log\left(1-f_{ON}\right)$$

## Supplementary Material



## Notes

Additional list of materials, reagents, and methods including gating strategy for singleplex and multiplex measurements, agarose gels, additional calibration curves, and raw data for plasma samples and multiplexed assays. (PDF)
