# Supplementary material for: Ultrasensitive Enzyme-Free Detection of Proteins on Magnetic Beads
Source: J Am Chem Soc. 2026 Mar 13;148(11):11641–53. doi: 10.1021/jacs.5c19560 (PMC13022858; doi:10.1021/jacs.5c19560)
Supplement: Supplementary file 1 [file ja5c19560_si_001.pdf]

Supporting Information

Ultrasensitive Enzyme-free Detection of Proteins on Magnetic Beads

Karan Malhotra[1,2,3], Louise L Hansen[1,2,3], Samuel Han[1,2,3], Shira Roth[1,2,3], David R. Walt[1,2,3]*

[1] Department of Pathology, Brigham and Women's Hospital, Harvard Medical School, Boston, MA, 02115, United States.
[2] Wyss Institute for Biologically Inspired Engineering, Harvard University, Boston, MA, 02215, United States.
[3] Harvard Medical School, Harvard University, Boston, MA, 02115, United States.

* Corresponding author, Email: dwalt@bwh.harvard.edu

Table of Contents

GATING WORKFLOW FOR SINGLEPLEX ASSAYS 3
GATING WORKFLOW FOR MULTIPLEX ASSAYS 4
VALIDATION OF HYBRIDIZATION CHAIN REACTION FOR GENERATING SIGNAL AMPLIFIERS 5
SOURCES OF BACKGROUND SIGNAL FROM NONSPECIFIC BINDING OF SIGNAL AMPLIFIERS 6
NON-BINDING VS. MEDIUM-BINDING 96-WELL PLATES 7
ALEXA647 VS. ATTO647 DYES ON HCR AMPLIFIERS 8
RAPID WORKFLOW FOR ACCELERATED READOUT BY EFMOSAIC 9
CALIBRATION CURVES FOR CYTOKINE PROTEINS USING SINGLE MOLECULE ARRAYS (SIMOA) 10
CALIBRATION CURVES FOR CYTOKINE PROTEINS USING EFMOSAIC 17
SIGNAL/BACKGROUND RATIO FOR SIMOA AND EFMOSAIC 25
VALIDATION OF EFMOSAIC WORKFLOW ON TWO ADDITIONAL FLOW CYTOMETERS 26
STREPTAVIDIN SIGNAL AMPLIFIER (SSA) STABILITY AT DIFFERENT STORAGE TEMPERATURES 27
DILUTION LINEARITY, AND SPIKE AND RECOVERY IN INDIVIDUAL HEALTHY HUMAN PLASMAS 28
MULTIPLEXED SIMOA ASSAYS 33
MULTIPLEXED EFMOSAIC ASSAYS 37
PHOTOBLEACHING DYE-ENCODED BEADS CAN IMPROVE SIGNAL/BACKGROUND RATIO 41
REAGENTS AND CONSUMABLES 43
SYNTHETIC OLIGONUCLEOTIDES 44
ANTIBODIES AND RECOMBINANT PROTEINS 45
BUFFERS RECIPES FOR THE EFMOSAIC ASSAY 46
Amplification Buffer 46
Wash Buffer 46
SUMMARY OF EFMOSAIC AND SIMOA ASSAY CONDITIONS 47
SUMMARY OF EFMOSAIC ASSAY WORKFLOW 48

Gating Workflow for Singleplex Assays

Beads were analyzed by flow cytometry. Once data was acquired for a sample, gating was done on the calibration blank (negative control) and applied to all samples, Figure S1. For a given singleplex measurement, gating was first done to select individual beads using the forward and side scatter plots. The gated population of beads were then analyzed for fluorescence signal (ATTO647, average fluorescence on beads in arbitrary units) and a histogram was generated for the bead population.

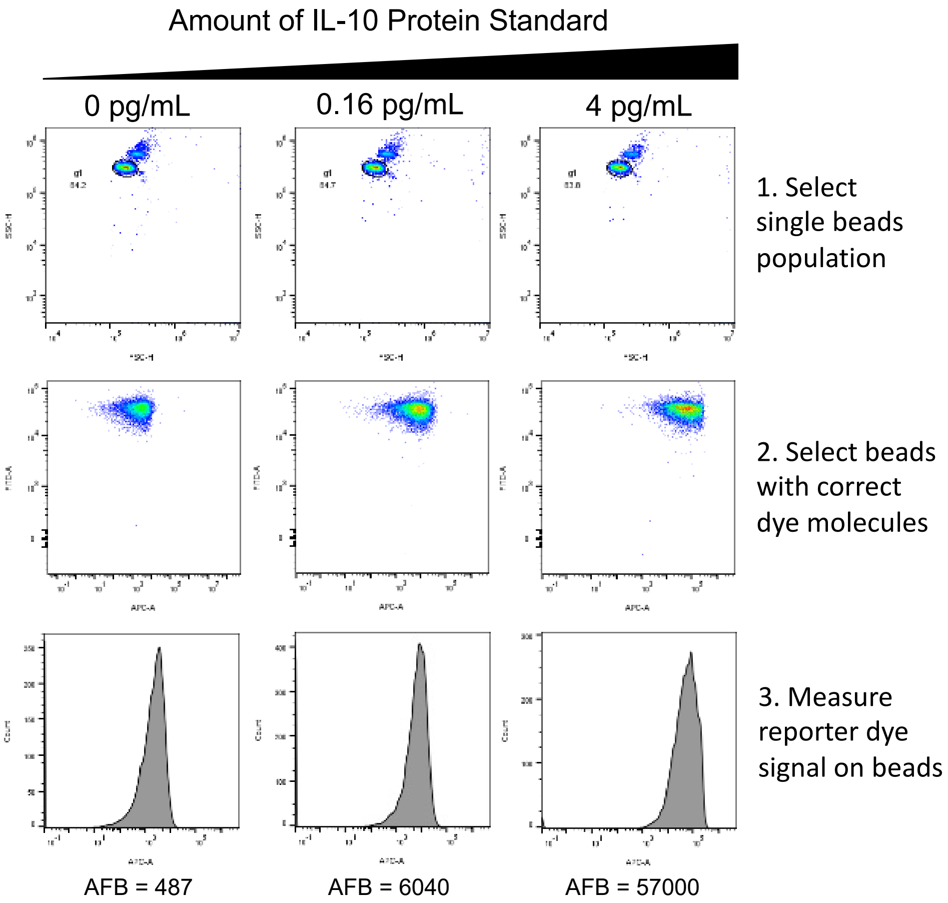

Figure S1. Overview of the gating strategy to measure reporter dye signal on beads. For a given sample well, gating was done in the forward and side scatter plot (FSC and SSC respectively) to pick single bead populations. Next, the beads were gated with 488 and 647 color channels. Here, the dye encoded beads were observed as a consistent population with an increasing amount of ATTO647 dye molecules on beads. The average fluorescence intensity on the entire bead population was used to generate a calibration curve for the corresponding protein standard.

Gating Workflow for Multiplex Assays

For multiplex assays, beads were analyzed in a similar manner to singleplex assays. Gating was done on the calibration blank (negative control) and applied to all samples in the acquisition, Figure S3. First, gating was done to select single beads using the forward and side scatter plots. The gated population of beads were then analyzed using the 488-color channel and side scatter channel to separate 488 beads from 750 beads. Next, the two populations of beads were analyzed for reporter signal (ATTO647, average fluorescence on beads in arbitrary units) and a histogram was generated for the two bead populations.

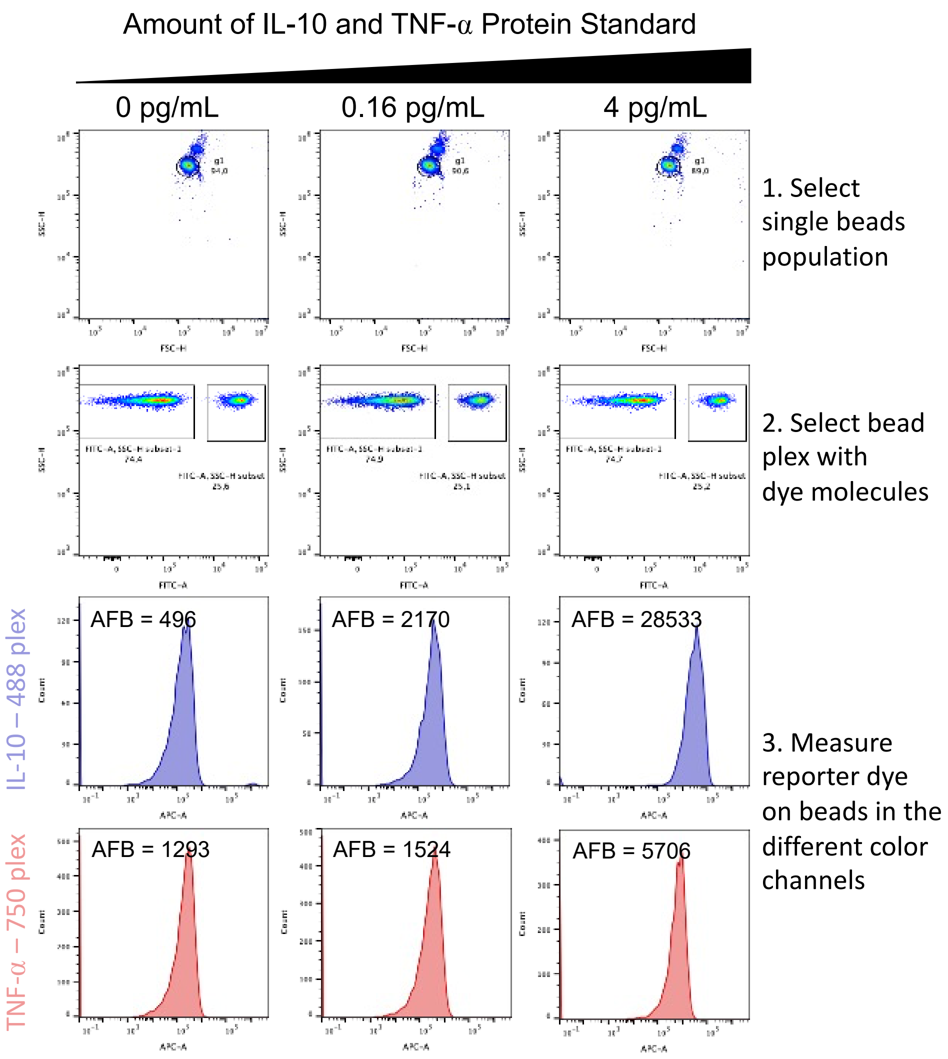

Figure S2. Overview of the gating strategy for the multiplexed measurement of IL-10 and TNF-α. For a given sample, gating was first done in the forward and side scatter plot (FSC and SSC respectively) to identify the population of single beads. Next, the beads were gated with 488-color and SSC channels to separate the dye-encoded beads into two populations. Once separated, signal in the ATTO647 channel was measured on beads. The average fluorescence intensity was measured and used to generate a calibration curve for the corresponding protein standard.

Validation of Hybridization Chain Reaction for Generating Signal Amplifiers

Streptavidin-conjugated signal amplifiers were generated using hybridization chain reaction (HCR). In HCR, two DNA hairpins coexist as independent species that do not interact with one another when trapped in kinetically stable conformations. The addition of an ***initiator*** oligonucleotide to the hairpins triggers toehold-mediated strand displacement resulting in the opening of one of the hairpins (H1). The resulting single-stranded domain of the open hairpin-initiator complex (I–H1) triggers the opening of the second hairpin (I–H1–H2). This series of reactions proceeds in an alternating manner between the two hairpins until all monomers are spent and an oligonucleotide-based signal amplifier is generated. DNA sequences for HCR were validated by performing *test tube* experiments for the conditional polymerization of hairpin amplifiers using custom oligonucleotides (Initiator Basic, H1 Basic, H2 Basic). A series of reaction mixtures were prepared, and gel electrophoresis was done on the reaction mixtures, Figure S3. Conditional polymerization by HCR was observed only in the presence of ***initiator*** and with decreasing amounts of ***initiator***, we observed larger constructs of the signal amplifiers.

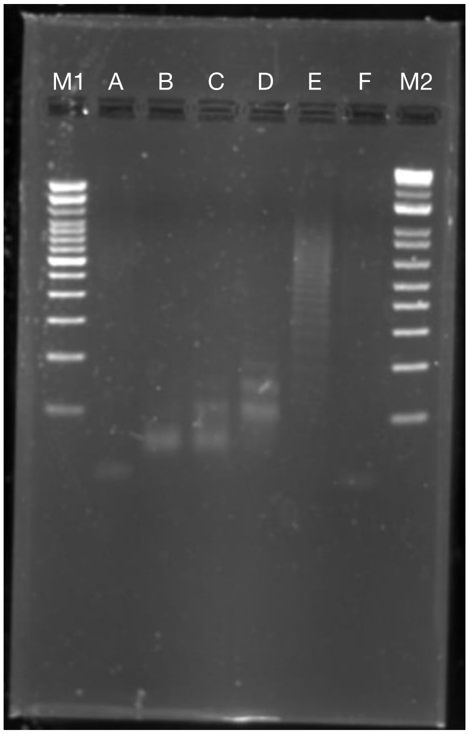

Figure S3. Agarose gel for products of the hybridization chain reaction. DNA amplifiers used to prepare the efMOSAIC SSA reagent were validated by gel electrophoresis. In the presence of *initiator*, the H1 and H2 hairpins underwent polymerization with an increasing mean length that correlated with decreasing amounts of *initiator* molecules. Four different *initiator* concentrations (B) 1×, (C) 0.1×, (D) 0.01×, (E) 0.001×, (F) 0.0001× were incubated with the H1 and H2 hairpins (1μM each). These mixtures were incubated for 1 hour and characterized with a Native 1% agarose gel (1×SB buffer) at 75V for 45 minutes. The gel was stained with SYBR Gold and imaged using the Sapphire Biomolecular Imager (Azure Biosystems, Dublin CA).

Sources of Background Signal from Nonspecific Binding of Signal Amplifiers

We quantified non-specific binding on beads by removing specific components from the amplifier reagent mixture, Figure S4:

a. No streptavidin-initiator and no hairpins.
b. No streptavidin-initiator and no H2 hairpins (H1 in solution).
c. No streptavidin-initiator (both H1 and H2 hairpins in solution).

In all cases, we did not observe any background signal due to nonspecific binding of SSA components to beads.

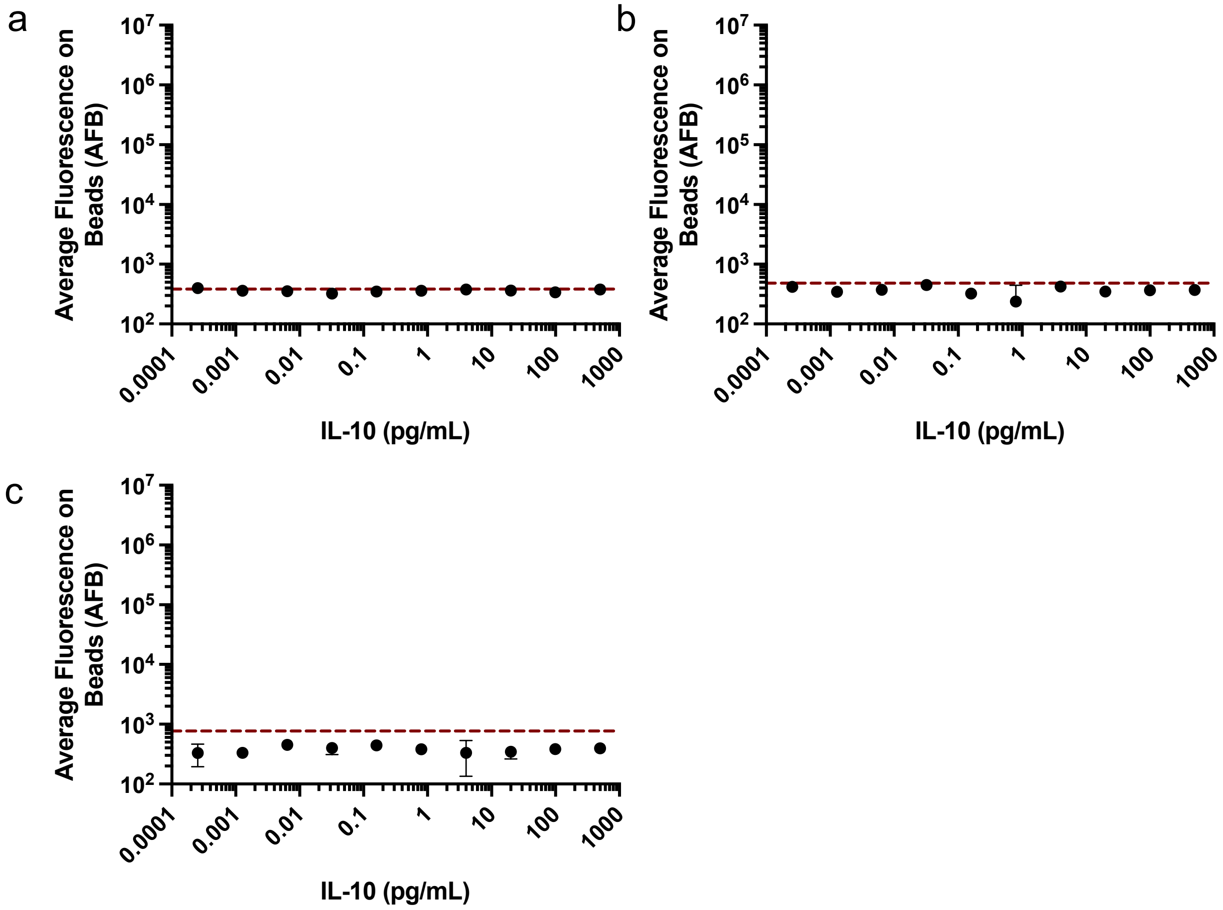

Figure S4. Calibration curves for the efMOSAIC assay of IL10 cytokine were done with variations of the SSA reagent to study the effects of nonspecific signal on the efMOSAIC assay. Here, the SSA mix had one of the following modifications: (a) No streptavidin-oligonucleotide conjugates and no hairpins, (b) No streptavidin-oligonucleotide conjugates and H1 hairpins only. (c) No streptavidin-oligonucleotide conjugates and both H1 and H2 hairpins. Error bars are standard deviation from two replicates for samples and four replicates for blank and dashed lines indicate the LOD.

Non-binding vs. Medium-binding 96-well plates

For initial testing, we investigated assay performance with non-binding and medium-binding plates. Non-binding 96-well assay plates are designed to minimize non-specific binding by reducing surface adsorption of biomolecules in well plates. Medium binding 96-well assay plates are designed to have some binding of biomolecules. In a head-to-head experiment, calibration curves of IL10 had negligible differences between the plates and non-binding coatings offered no advantage over the medium binding plates. We believe that excess bovine serum albumin protein in the sample diluent minimized loss of target proteins to the well plates and as a result, we see no significant change in assay performance.

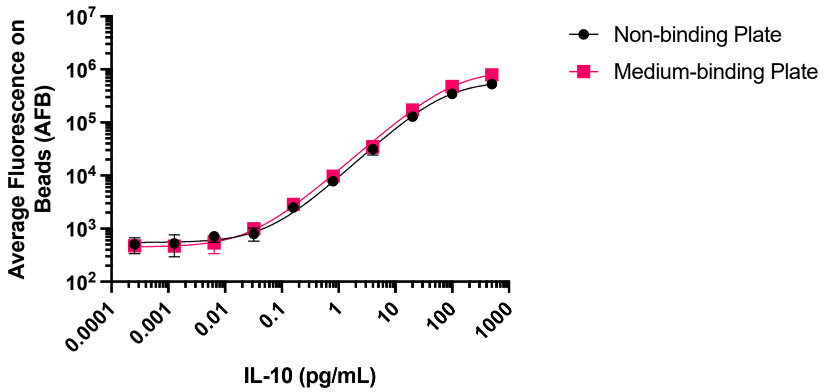

Figure S5. Calibration Curves for the efMOSAIC assay of IL10 cytokine using nonbinding (LOD = 0.016 pg/mL) and medium binding plates (0.009 pg/mL). Curves were fitted using the four-parameter logistic regression (4PL). Error bars were standard deviation from three replicates for samples and six replicates for blank.

Alexa647 vs. ATTO647 Dyes on HCR Amplifiers

efMOSAIC assay performance is dependent in part on the photoluminescence properties of the fluorescent dye molecules that are used to generate SSA reagent. There are several commercially available dyes that may be used to label DNA hairpins including Alexa fluor and ATTO-TEC dyes. We tested the signal generated from signal amplifiers conjugated to Alexa647, or ATTO647, Figure S6.

We found that ATTO647 dyes generated slightly better signal for a given concentration than the Alexa647 dye molecules. Therefore, we used ATTO647 modified hairpins for all other work.

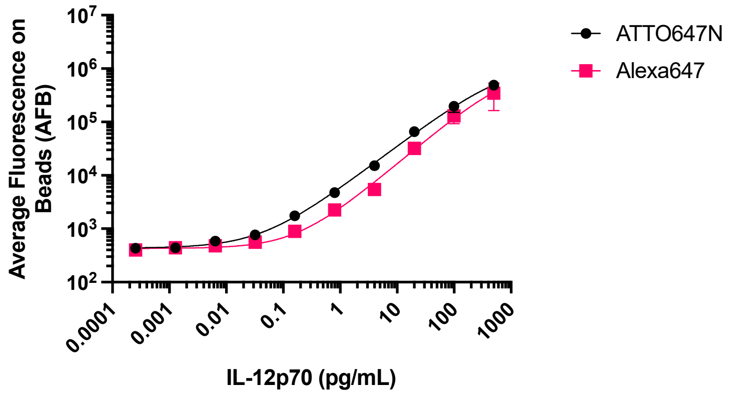

Figure S6. Calibration Curve for the efMOSAIC assay of IL12p70 protein using ATTO647N Dye conjugated hairpins (LOD = 0.011 pg/mL) and Alexa647 Dye conjugated hairpins (LOD = 0.054 pg/mL). Curves were fitted using the four-parameter logistic regression (4PL). Error bars are standard deviation from three replicates for samples and six replicates for blank.

Rapid Workflow for Accelerated Readout by efMOSAIC

We investigated the performance of a rapid workflow for efMOSAIC with shorter incubations times and wash steps, Table S1. Calibration curves for the normal and rapid formats of efMOSAIC are given in Figure S7.

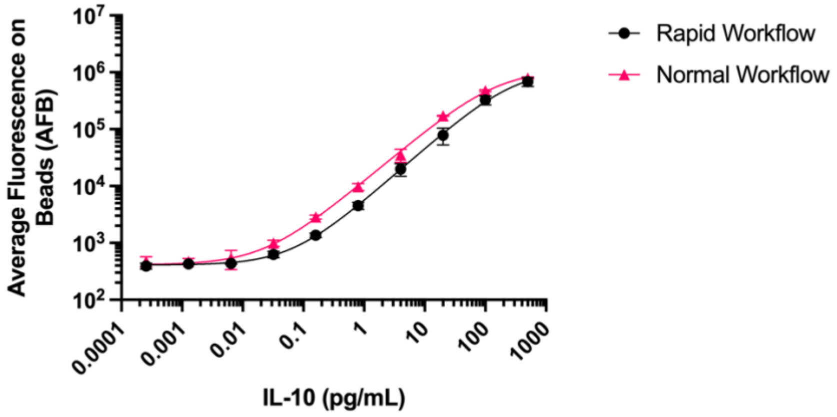

Figure S7. Calibration Curve for the efMOSAIC assay of IL10 protein with the normal and rapid workflows. Curves were fitted using the four-parameter logistic regression (4PL). Error bars are standard deviation from three replicates for samples and six replicates for blank.

Table S1. Experimental Conditions for Different efMOSAIC Assay Workflows

	Normal Workflow	Rapid Workflow
Incubation Times (mins/step)	1 hour 45 minutes (60-15-30)	1 hour (15-15-30)
Wash Times (6 = 6 washes, 3 = 3 washes)	~ 40 minutes (6-6-3)	~ 30 minutes (3-3-3)
Total Assay Time	~ 2 hour 25 minutes	~ 1 hour 30 minutes
LOD	9 fg/mL	12 fg/mL
LLOQ	43 fg/mL	39 fg/mL

Calibration Curves for Cytokine Proteins using Single Molecule Arrays (Simoa)

To benchmark the performance of efMOSAIC assays, we ran a set of complementary Simoa assays using the same batch of beads, protein standards, and detector antibodies as efMOSAIC. Each set of calibration curves was run on a different day, Figure S8 – S14 and Table S2.

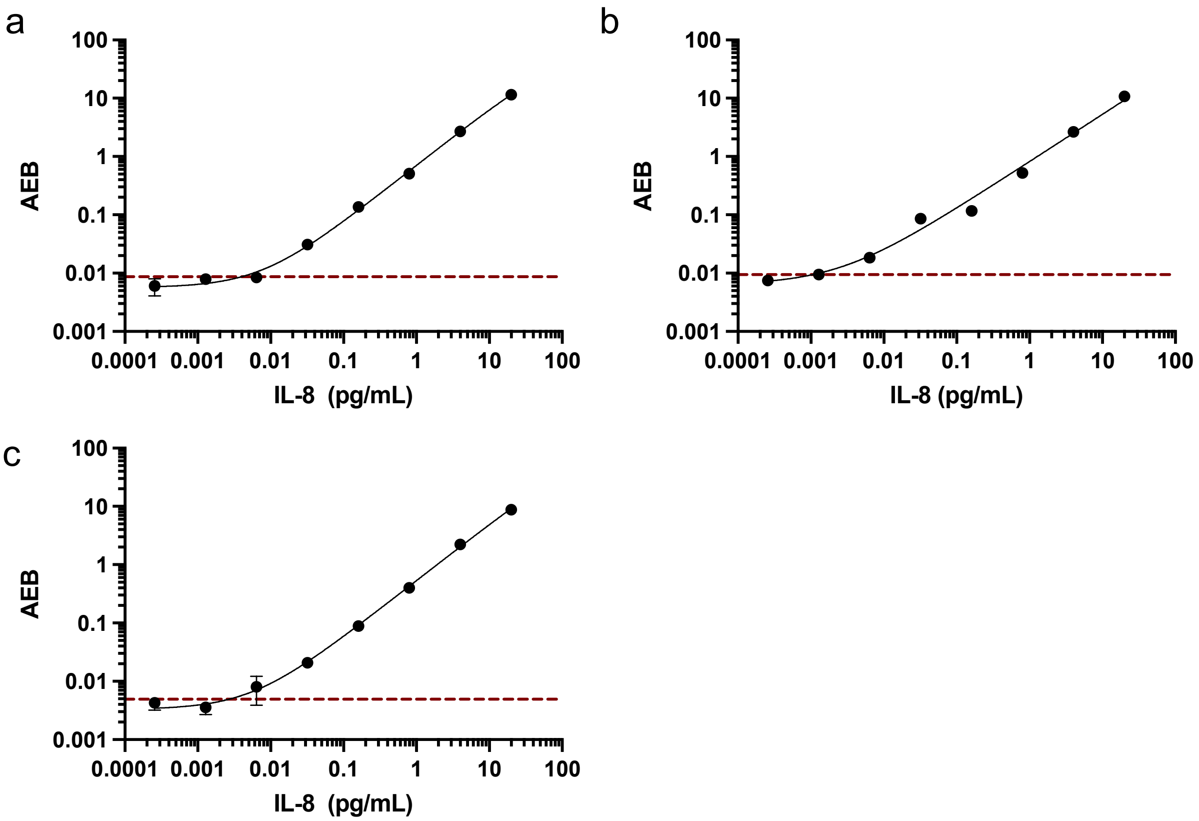

Figure S8. Simoa calibration curves for IL-8 protein in sample diluent was done on three different days. Here, AEB denotes the average enzyme on beads, and the sensitivities for these assays were (a) LOD = 4 fg/mL, (b) LOD = 1 fg/mL, and (c) LOD = 3 fg/mL respectively. Error bars are standard deviation from three replicates for samples and blanks. Curves were fitted using the four-parameter logistic regression (4PL) and dashed lines indicate the LOD.

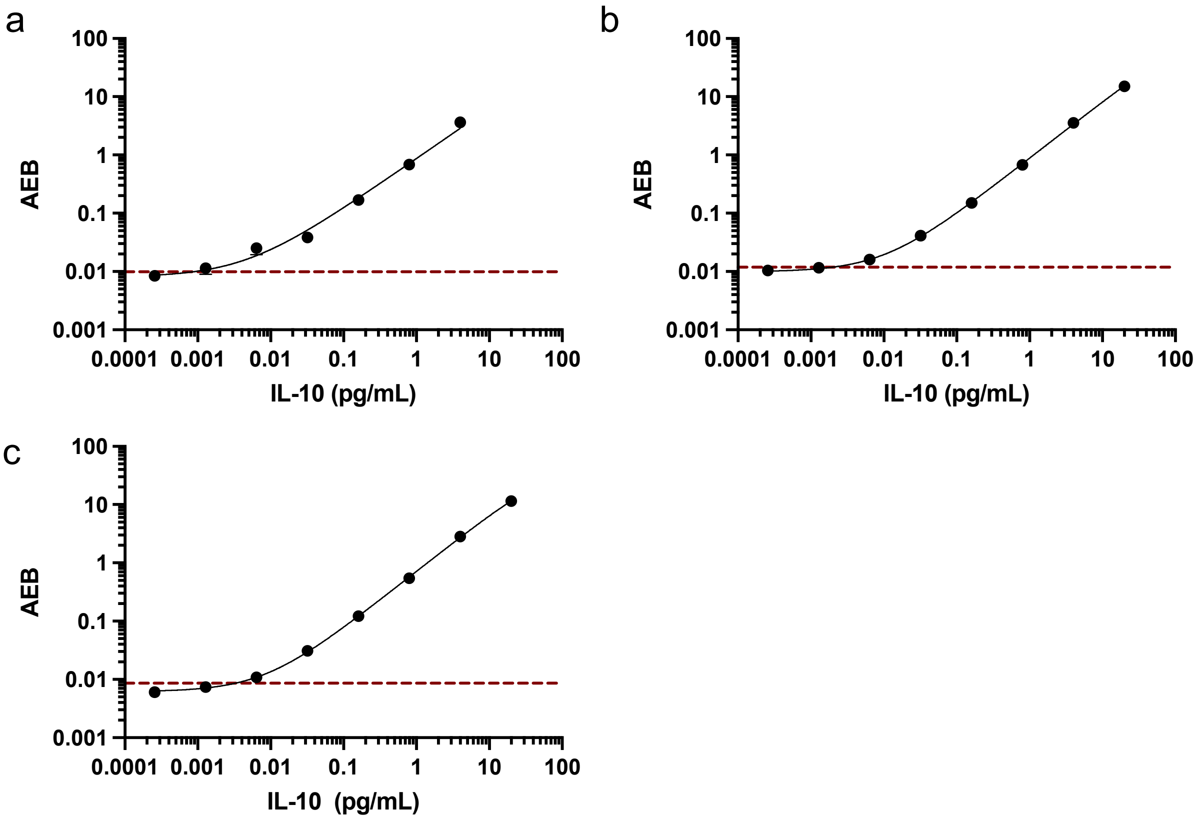

Figure S9. Simoa calibration curves for IL-10 protein in sample diluent was done on three different days. Here, AEB denotes the average enzyme on beads, and the sensitivities for these assays were (a) LOD = 1 fg/mL, (b) LOD = 2 fg/mL, and (c) LOD = 3 fg/mL respectively. Error bars are standard deviation from three replicates for samples and blanks. Curves were fitted using the four-parameter logistic regression (4PL) and dashed lines indicate the LOD.

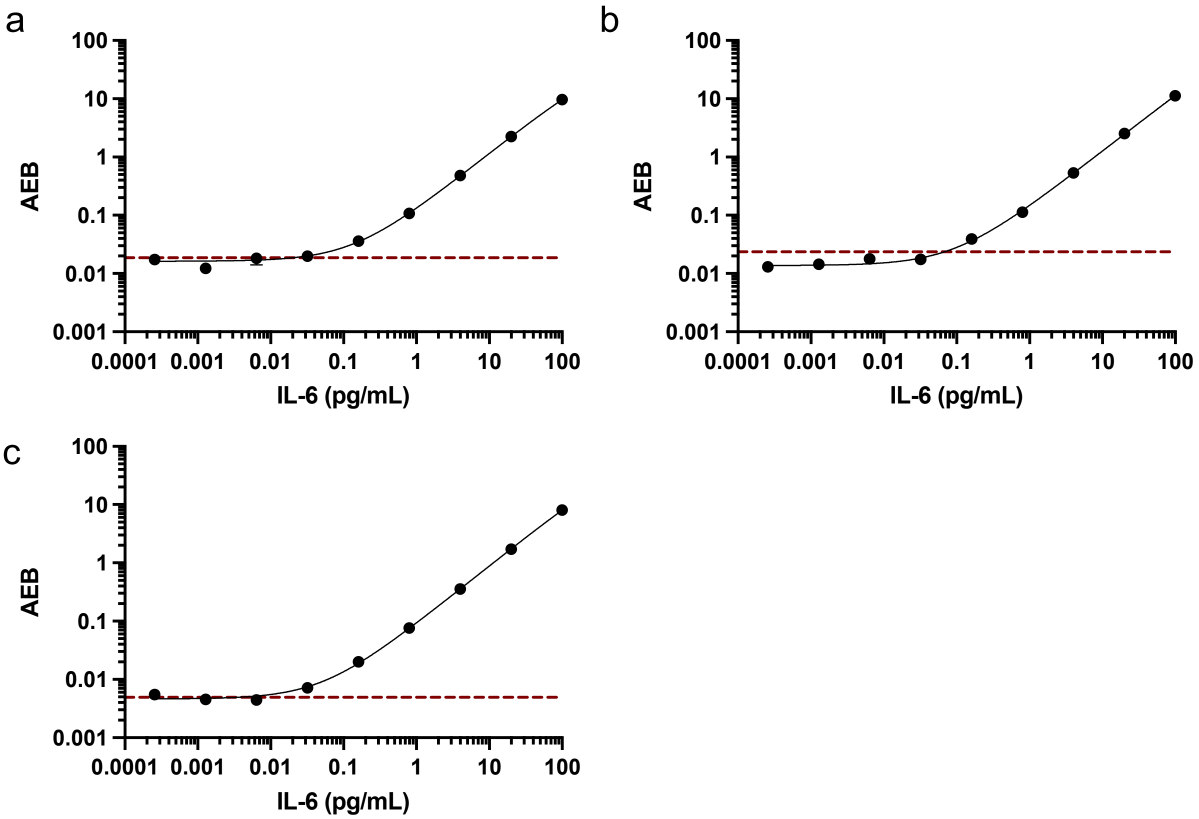

Figure S10. Simoa calibration curves for IL-6 protein in sample diluent was done on three different days. Here, AEB denotes the average enzyme on beads, and the sensitivities for these assays were (a) LOD = 20 fg/mL, (b) LOD = 68 fg/mL, and (c) LOD = 3 fg/mL respectively. Error bars are standard deviation from three replicates for samples and blanks. Curves were fitted using the four-parameter logistic regression (4PL) and dashed lines indicate the LOD.

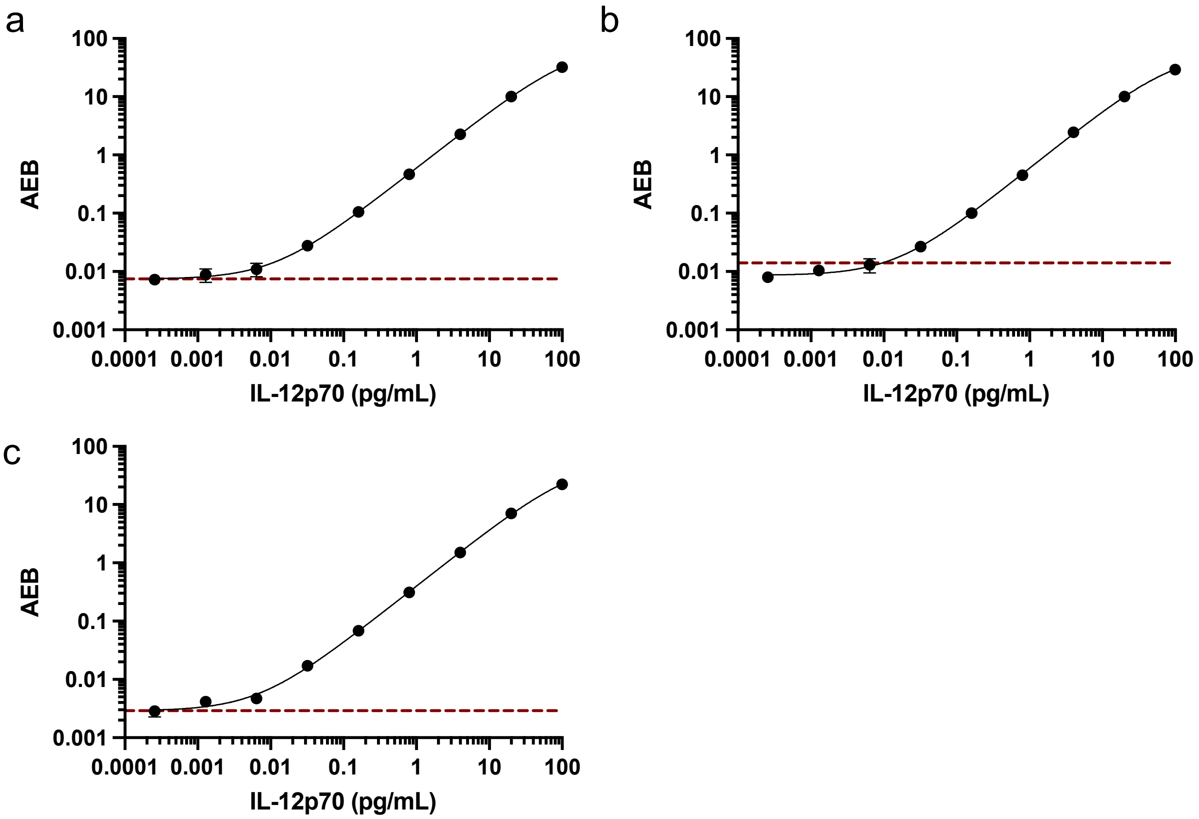

Figure S11. Simoa calibration curves for IL-12p70 protein in sample diluent was done on three different days. Here, AEB denotes the average enzyme on beads, and the sensitivities for these assays were (a) LOD = 1 fg/mL, (b) LOD = 9 fg/mL, and (c) LOD = 1 fg/mL respectively. Error bars are standard deviation from three replicates for samples and blanks. Curves were fitted using the four-parameter logistic regression (4PL) and dashed lines indicate the LOD.

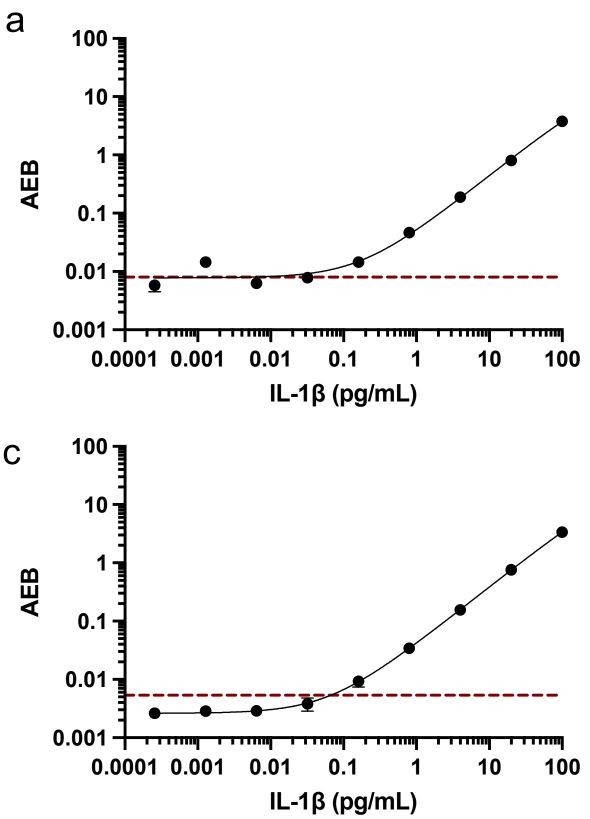

Figure S12. Simoa calibration curves for IL-1β protein in sample diluent was done on three different days. Here, AEB denotes the average enzyme on beads, and the sensitivities for these assays were (a) LOD = 5 fg/mL, (b) LOD = 147 fg/mL, and (c) LOD = 66 fg/mL respectively. Error bars are standard deviation from three replicates for samples and blanks. Curves were fitted using the four-parameter logistic regression (4PL) and dashed lines indicate the LOD.

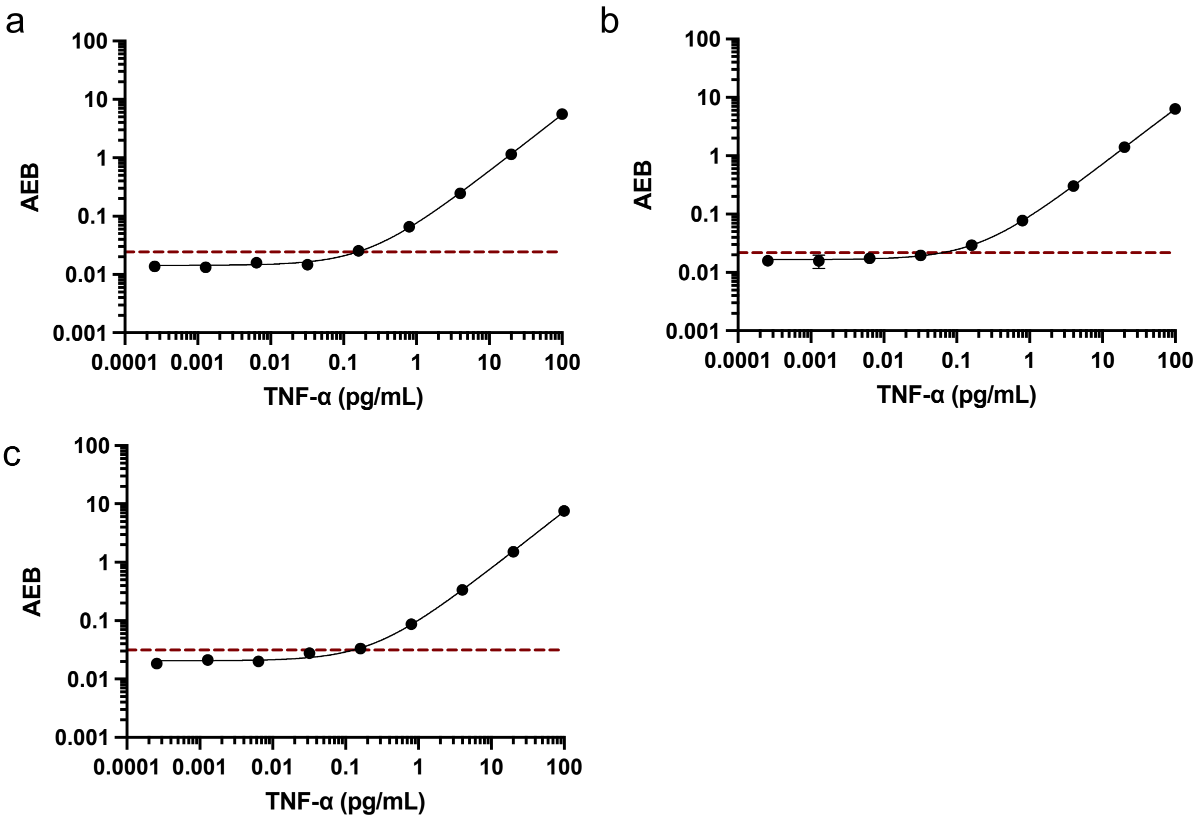

Figure S13. Simoa calibration curves for TNF-α protein in sample diluent was done on three different days. Here, AEB denotes the average enzyme on beads, and the sensitivities for these assays were (a) LOD = 153 fg/mL, (b) LOD = 63 fg/mL, and (c) LOD = 127 fg/mL respectively. Error bars are standard deviation from three replicates for samples and blanks. Curves were fitted using the four-parameter logistic regression (4PL) and dashed lines indicate the LOD.

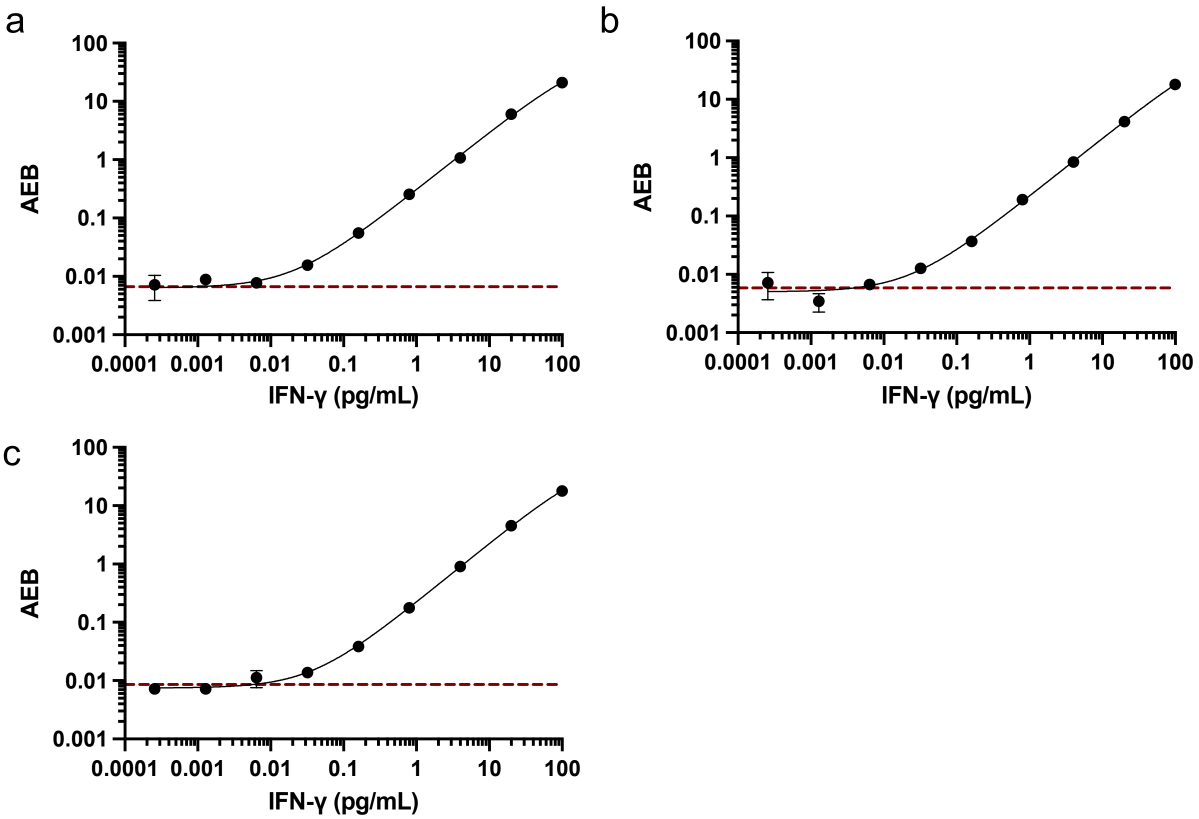

Figure S14. Simoa calibration curves for IFN-γ protein in sample diluent was done on three different days. Here, AEB denotes the average enzyme on beads, and the sensitivities for these assays were (a) LOD = 1 fg/mL, (b) LOD = 4 fg/mL, and (c) LOD = 6 fg/mL respectively. Error bars are standard deviation from three replicates for samples and blanks. Curves were fitted using the four-parameter logistic regression (4PL) and dashed lines indicate the LOD.

Table S2. Summary of the LODs and LLOQs for Cytokine Proteins by Simoa

Protein Target	Limit of Detection (fg/mL)			Lower Limit of Quantification (fg/mL)		
	1	2	3	1	2	3
IL-8	4	1	3	14	5	12
IL-10	1	2	3	5	8	10
IL-6	20	68	3	33	250	11
IL-12p70	1	9	1	1	34	1
IL-1β	5	147	66	67	488	232
TNF-α	153	63	127	462	187	399
IFN-γ	1	4	6	21	20	30

*LOD and LLOQ are measured as three and 10 SD above noise, respectively. Each set of replicates was done on a different day.

Calibration Curves for Cytokine Proteins using efMOSAIC

efMOSAIC assays were developed for cytokine proteins and independent replicates of the calibration curves were acquired on three different days, Figure S15 – S20, Table S3 and S4.

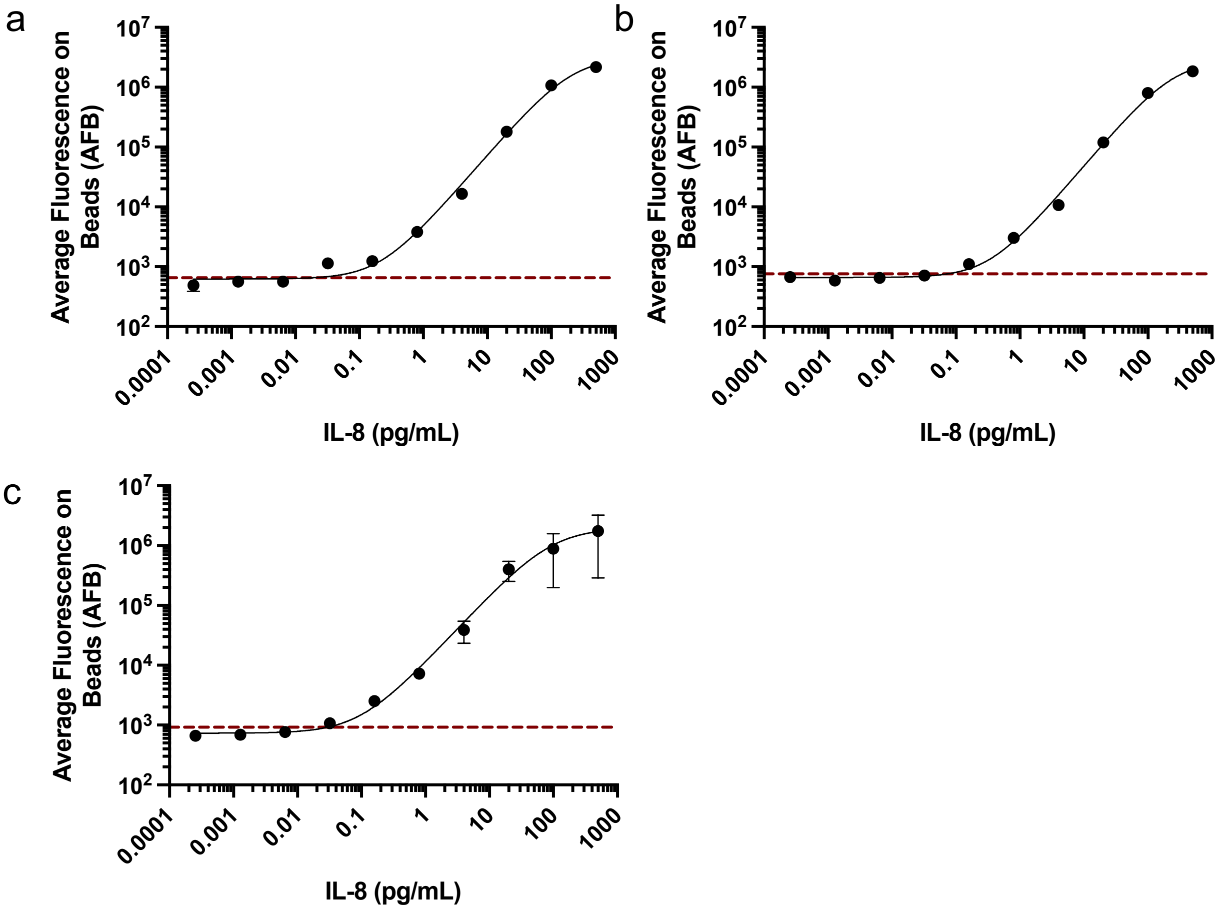

Figure S15. efMOSAIC calibration curves for IL-8 protein in sample diluent was done on three different days. Here, AFB denotes the average fluorescence on beads, and the sensitivities for these assays were (a) LOD = 18 fg/mL, (b) LOD = 12 fg/mL, and (c) LOD = 29 fg/mL respectively. Error bars are standard deviation from three replicates for samples and blanks. Curves were fitted using the four-parameter logistic regression (4PL) and dashed lines indicate the LOD.

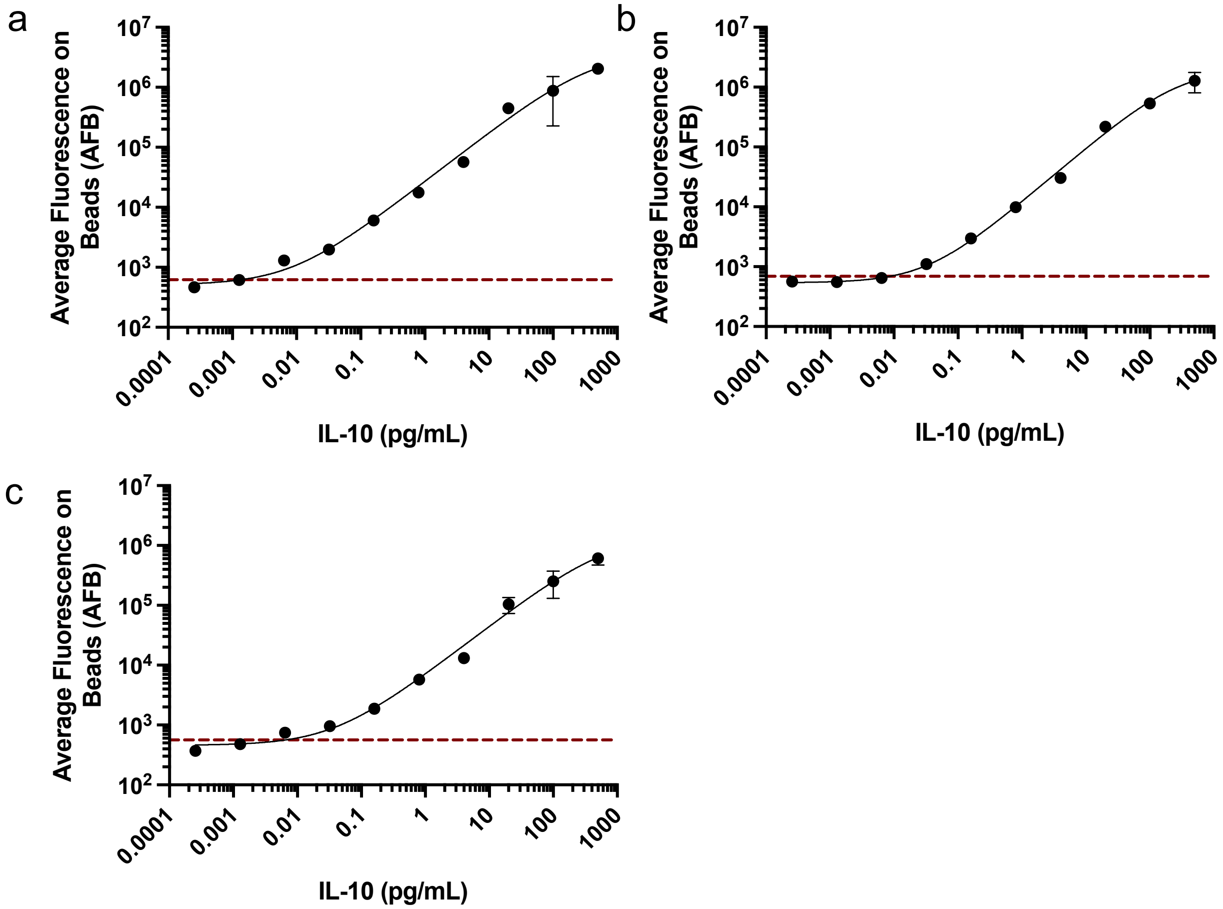

Figure S16. efMOSAIC calibration curves for IL-10 protein in sample diluent was done on three different days. Here, AFB denotes the average fluorescence on beads, and the sensitivities for these assays were (a) LOD = 1 fg/mL, (b) LOD = 9 fg/mL, and (c) LOD = 6 fg/mL respectively. Error bars are standard deviation from three replicates for samples and blanks. Curves were fitted using the four-parameter logistic regression (4PL) and dashed lines indicate the LOD.

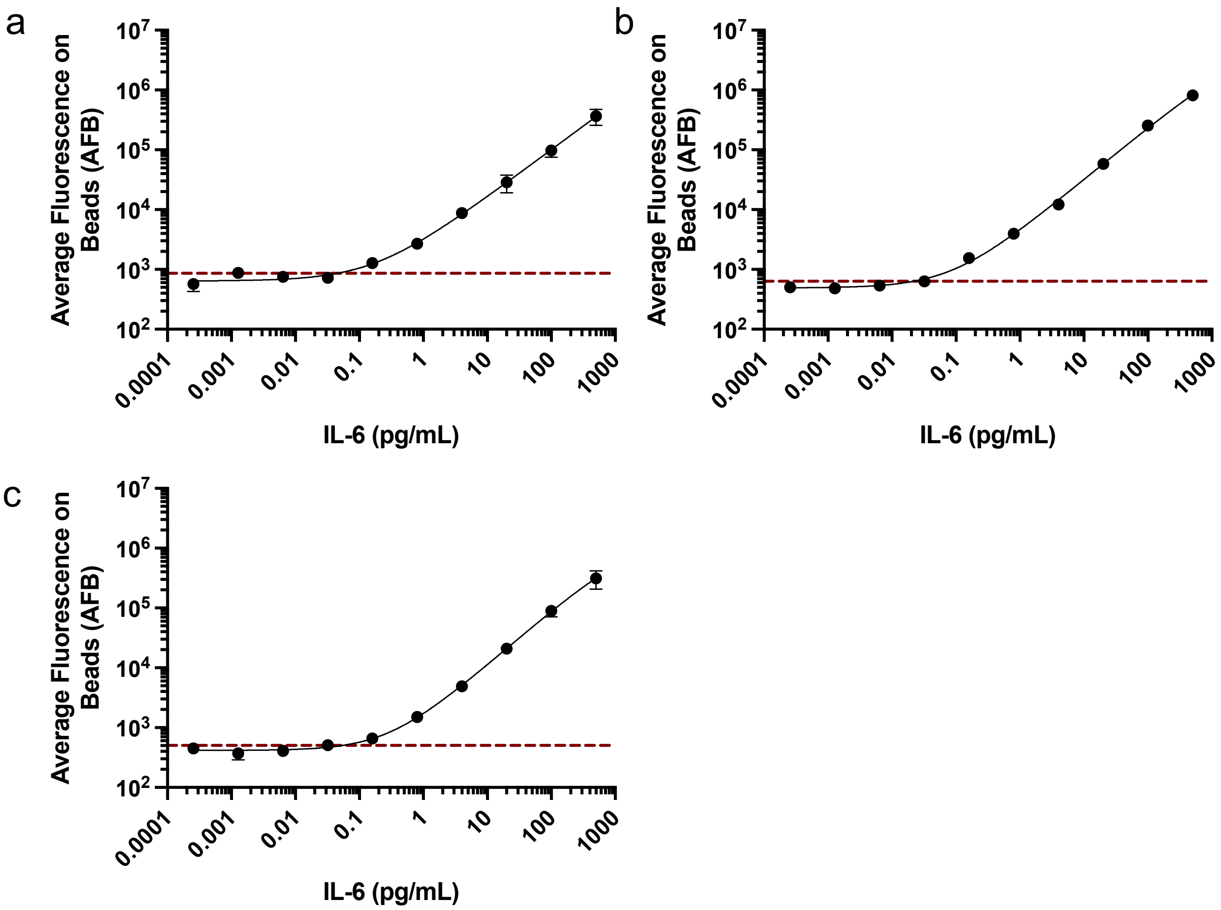

Figure S17. efMOSAIC calibration curves for IL-6 protein in sample diluent was done on three different days. Here, AFB denotes the average fluorescence on beads, and the sensitivities for these assays were (a) LOD = 45 fg/mL, (b) LOD = 21 fg/mL, and (c) LOD = 54 fg/mL respectively. Error bars are standard deviation from three replicates for samples and blanks. Curves were fitted using the four-parameter logistic regression (4PL) and dashed lines indicate the LOD.

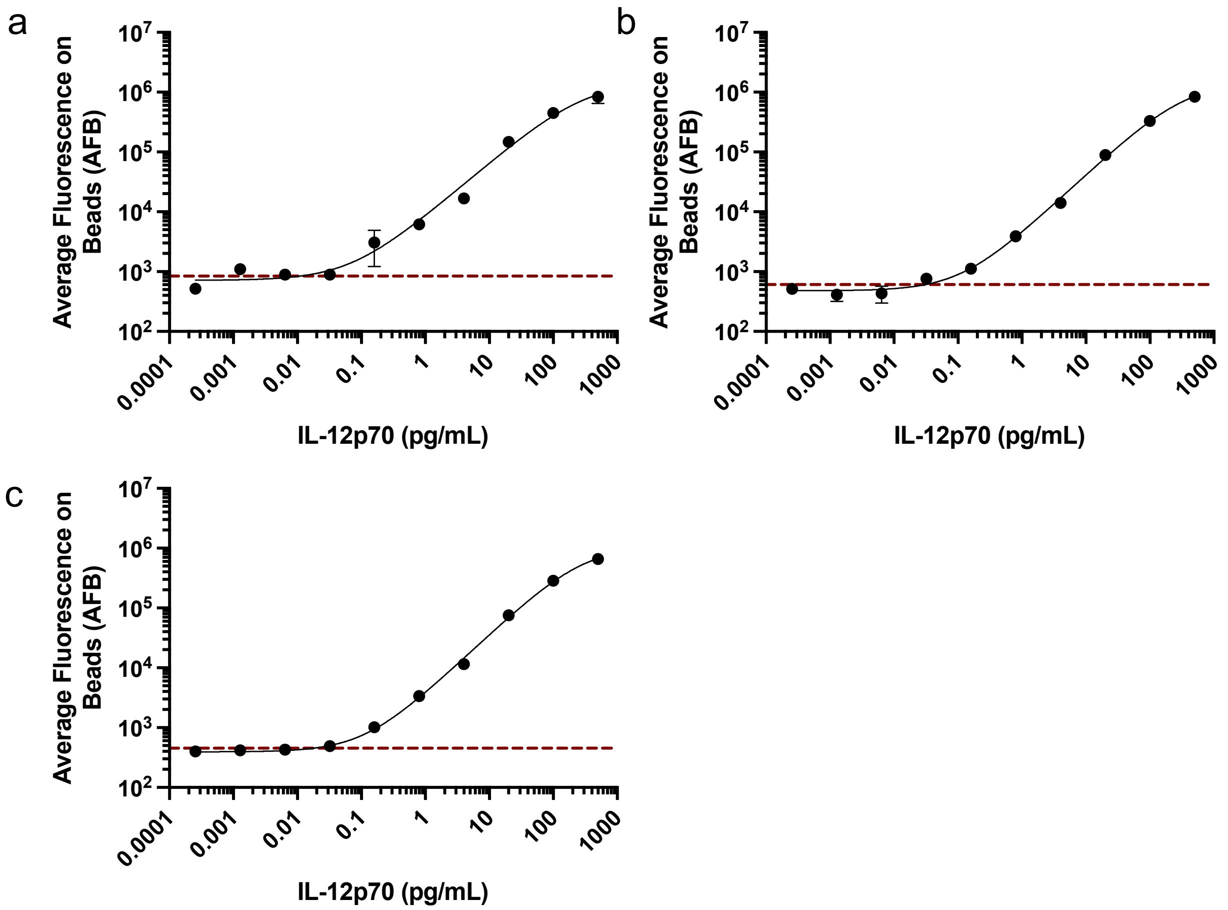

Figure S18. efMOSAIC calibration curves for IL-12p70 protein in sample diluent was done on three different days. Here, AFB denotes the average fluorescence on beads, and the sensitivities for these assays were (a) LOD = 11 fg/mL, (b) LOD = 31 fg/mL, and (c) LOD = 17 fg/mL respectively. Error bars are standard deviation from three replicates for samples and blanks. Curves were fitted using the four-parameter logistic regression (4PL) and dashed lines indicate the LOD.

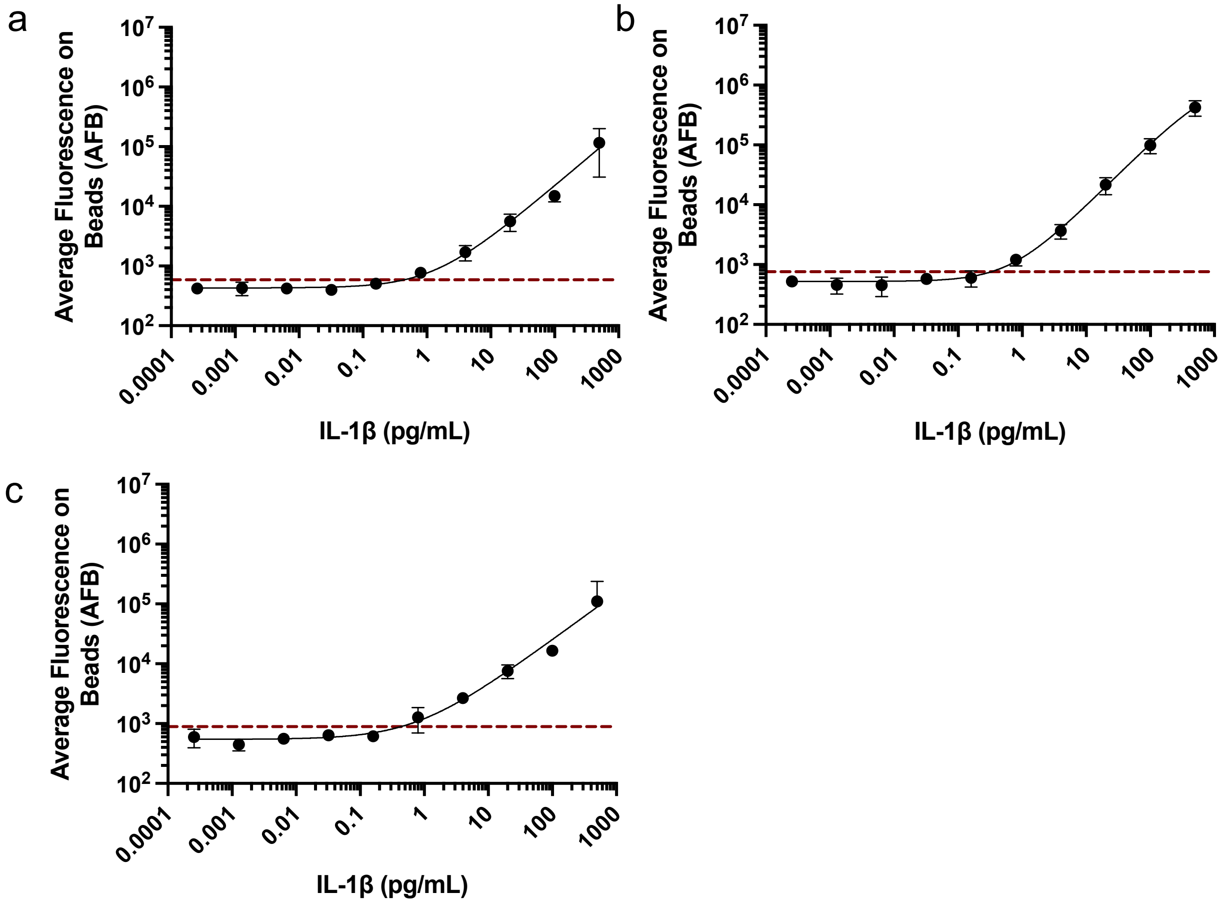

Figure S19. efMOSAIC calibration curves for IL-1β protein in sample diluent was done on three different days. Here, AFB denotes the average fluorescence on beads, and the sensitivities for these assays were (a) LOD = 426 fg/mL, (b) LOD = 329 fg/mL, and (c) LOD = 336 fg/mL respectively. Error bars are standard deviation from three replicates for samples and blanks. Curves were fitted using the four-parameter logistic regression (4PL) and dashed lines indicate the LOD.

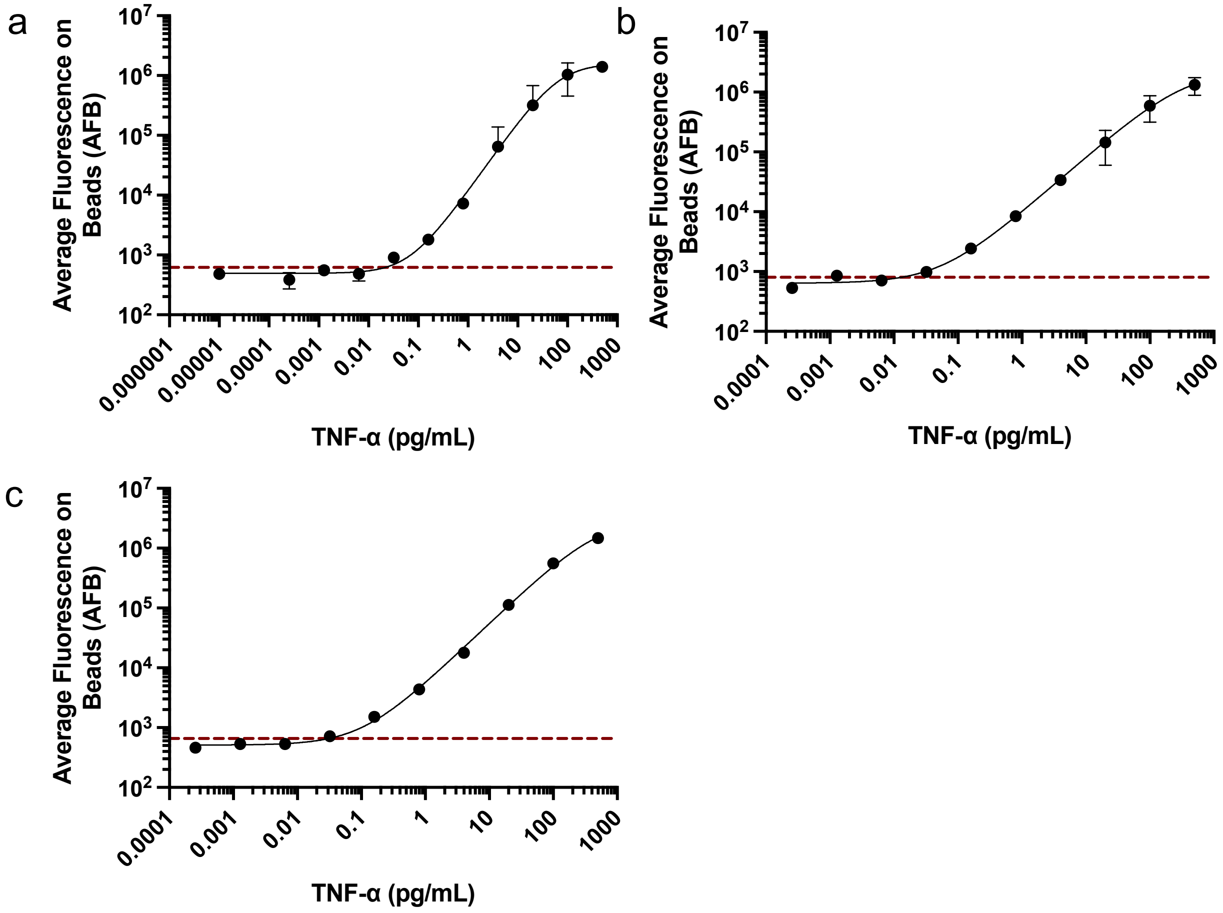

Figure S20. efMOSAIC calibration curves for TNF-α protein in sample diluent was done on three different days. Here, AFB denotes the average fluorescence on beads, and the sensitivities for these assays were (a) LOD = 31 fg/mL, (b) LOD = 21 fg/mL, and (c) LOD = 13 fg/mL respectively. Error bars are standard deviation from three replicates for samples and blanks. Curves were fitted using the four-parameter logistic regression (4PL) and dashed lines indicate the LOD.

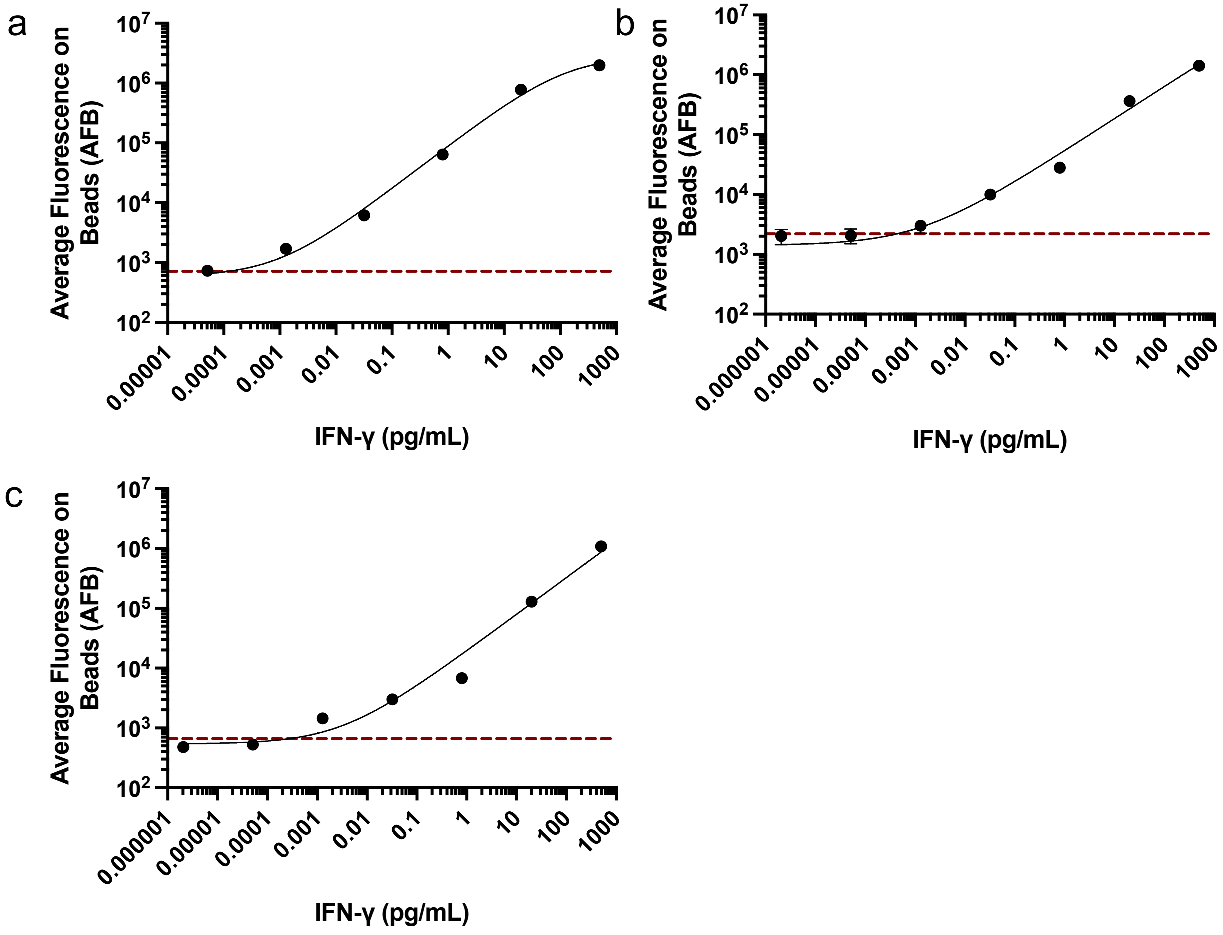

Figure S21. efMOSAIC calibration curves for IFN-γ protein in sample diluent was done on three different days. Here, AFB denotes the average fluorescence on beads, and the sensitivities for these assays were (a) LOD = 0.1 fg/mL, (b) LOD = 0.1 fg/mL, and (c) LOD = 0.2 fg/mL respectively. Error bars are standard deviation from three replicates for samples and blanks. Curves were fitted using the four-parameter logistic regression (4PL) and dashed lines indicate the LOD.

Table S3. Summary of the LODs and LLOQs for Proteins using efMOSAIC

Protein Target	Limit of Detection (fg/mL)			Lower Limit of Quantification (fg/mL)		
	1	2	3	1	2	3
IL-8	18	12	29	174	109	86
IL-10	1	9	6	8	36	39
IL-6	45	21	54	335	78	206
IL-12p70	11	31	17	98	128	85
IL-1β	426	329	336	1898	683	1151
TNF-α	31	21	13	108	63	38
IFN-γ	0.1	0.1	0.2	1	1	3

*LOD and LLOQ are measured as 3 and 10 SD above noise, respectively. Each set of replicates was done of a different day.

Table S4. Comparison of the range of LODs and LLOQs for Simoa and efMOSAIC Assays

Analyte	Limit of Detection (fg/mL)		Limit of Quantification (fg/mL)	
	efMOSAIC	Simoa	efMOSAIC	Simoa
IL-8	12 – 29	1 – 4	86 – 174	5 – 14
IL-10	1 – 9	1 – 3	8 – 39	5 – 10
IL-6	21 – 45	3 – 68	78 – 335	11 – 250
IL-12p70	11 – 31	1 – 9	85 – 128	1 – 34
IL-1β	329 – 426	5 – 147	683 – 1898	67 – 488
TNF-α	13 – 31	63 – 153	38 – 108	187 – 462
IFN-γ	0.1 – 0.2	1 – 6	1 – 3	20 – 30

Signal/Background Ratio for Simoa and efMOSAIC

We determined the signal/background ratios for each of the calibration curves generated by Simoa and efMOSAIC in this report (Figures S22). For most assays, except TNF-α and IFN-γ, Simoa was found to have slightly better or equal performance than the corresponding efMOSAIC assay.

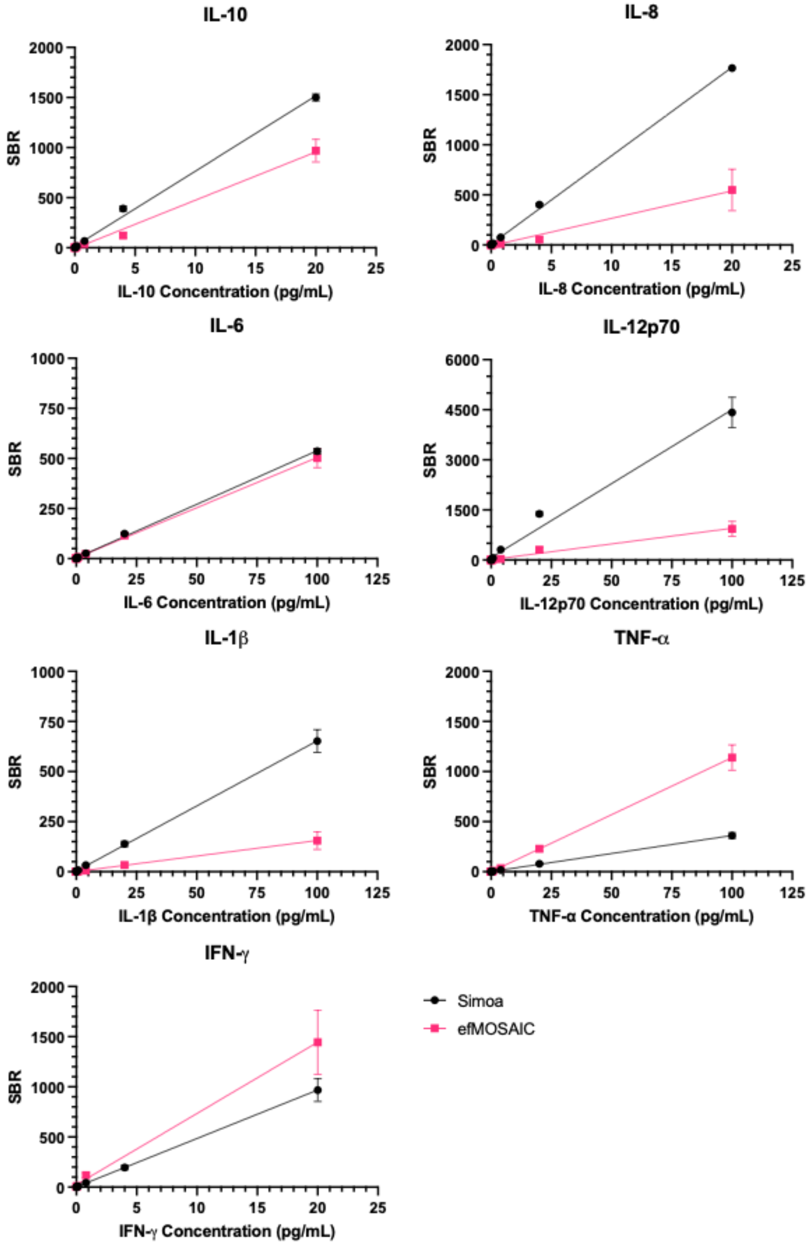

Figure S22. Comparison of the signal/background ratio (SBR) for Simoa and efMOSAIC for the Singleplex IL-10, IL-8, IL-6, IL-12p70, IL-1β, TNF-α, and IFN-γ assays. Error bars are standard deviation from three replicates for samples and blanks, and curves were fitted using simple linear regression.

Validation of efMOSAIC Workflow on Two Additional Flow Cytometers

We tested the performance of the efMOSAIC workflow with two additional flow cytometers, the Beckman Coulter Cytoflex S and Cytoflex LX. The Cytoflex S is a robust entry-level flow cytometer sold by Beckman Coulter and it is well suited for use in low-resource settings. It has a small size footprint and lighter weight when compared to other research flow cytometers. For example, when compared to the Agilent Novocyte 3000RYB flow cytometer used in most of our work, the Cytoflex S is 38% smaller and 41% lighter. The simple design of the Cytoflex S makes it robust for use in resource-limited settings. We ran a calibration curve for the IL-10 protein (Figure S23) using the same beads, detector antibodies, and protein standards as our other experiments. We found that the efMOSAIC assay for IL-10 performed equally well to IL-10 assays that were run on the Agilent Novocyte 3000RYB (main text Figure 3 and Table 1) and the Cytoflex LX systems. Our data suggests that the efMOSAIC workflow should be compatible with many different flow cytometers for readout.

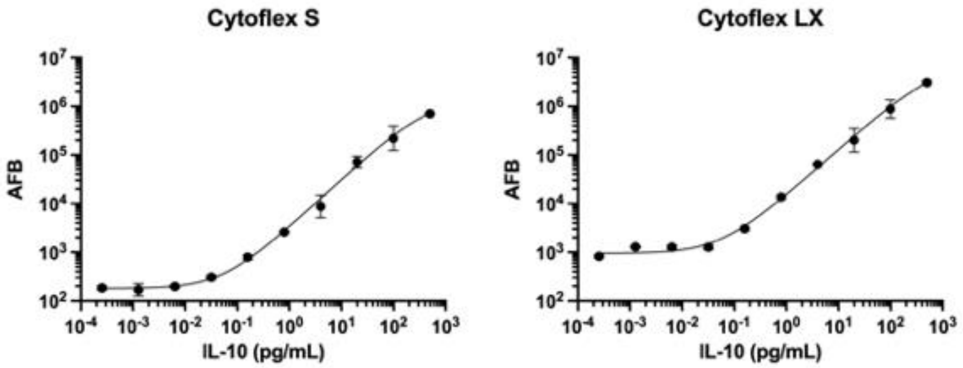

Figure S23. Comparison of performance for the efMOSAIC assay workflow on two additional flow cytometers. Calibration curves for IL-10 were done in the singleplex format. All calibration curves were fitted using the four-parameter logistic (4PL) regression and error bars were standard deviations from three replicates.

Table S5. Comparison of the LOD and LLOQ of the IL-10 efMOSAIC Assay on Two Commercially Available Benchtop Flow Cytometers

Flow Cytometer	LOD	LLOQ
Beckman Coulter Cytoflex S	5 fg/mL	18 fg/mL
Beckman Coulter Cytoflex LX	1 fg/mL	33 fg/mL

Streptavidin Signal Amplifier (SSA) Stability at Different Storage Temperatures

SSA reagent stability was investigated at different temperatures (– 20°C, 4°C and 21°C) by generating a calibration curve for IL-10. The storage conditions and the corresponding assay sensitivities in the main manuscript as Figure 3(c), Figure S23 and Table S5. In general, no loss in sensitivity or assay performance was observed for SSA reagent stored at the various temperature conditions.

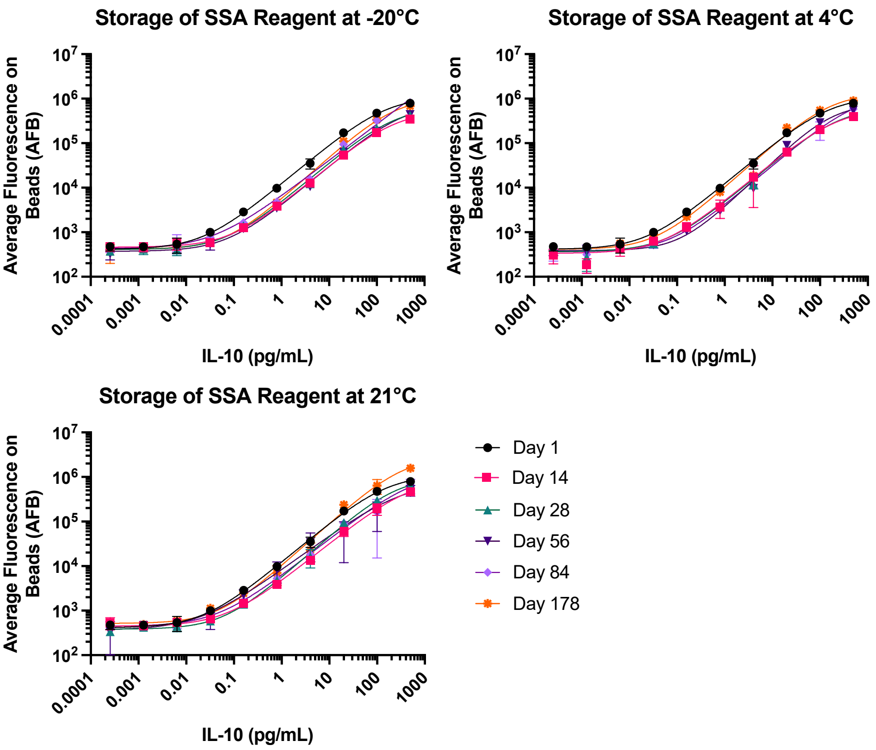

Figure S24. SSA Reagent stability was investigated for – 20°C, 4°C, and 21°C storage temperatures. In general, the reagent was stable over 178 days of storage (6 months) for all conditions investigated. Error bars are standard deviation from three replicates for samples and blanks. Curves were fitted using the four-parameter logistic regression (4PL).

Table S6. Storage Conditions for the IL-10 efMOSAIC Assay

Storage Time (days)	Limit of Detection (fg/mL)*			Lower Limit of Quantification (fg/mL)*		
	-20°C	4°C	21°C	-20°C	4°C	21°C
0	9			43		
14	61	43	34	245	134	163
28	16	15	52	50	73	186
56	27	57	9	100	183	36
84	25	40	37	113	150	148
178	23	14	19	81	57	69

*LOD and LLOQ are measured as 3 and 10 SD above noise, respectively.

Dilution Linearity, and Spike and Recovery in Individual Healthy Human Plasmas

efMOSAIC was validated by measuring endogenous protein concentration in plasma samples from three different individuals by dilution linearity (parallelism). Plasma was serially diluted to obtain a set of test samples (from 2-fold to 32-fold dilution). Samples were then measured by efMOSAIC and processed to determine the average amount of endogenous protein in each assay. Separately, the percent linearity was calculated using Equation S1. In general, an assay was found to have good linearity if the measured concentration was within a range of 80 – 120 % of the expected concentration (as determined by the previous concentration). A summary of these concentrations and percent linearity (% Linearity) is given as Table S6. Additionally, the corresponding dilution linearity trendlines were determined for each assay and presented as Figure S24, and Tables S6 – S13.

Equation S1. $\textbf{Percent Linearity}\ (\%) = \textbf{Dilution Fold} \times \frac{\textbf{Observed Concentration}}{\textbf{Previous Concentration}} \times \mathbf{100}\ \%$

Table S7. Validation of efMOSAIC assay with parallelism of proteins in individual human plasma samples.

Singleplex efMOSAIC Assays	Average Amount of Endogenous Proteins in Individual Plasmas (pg/mL)		
	Plasma 1	Plasma 2	Plasma 3
IL-8	20.00 ± 1.50	12.73 ± 0.65	19.92 ± 0.54
IL-10	0.12 ± 0.01	0.20 ± 0.01	0.09 ± 0.01
IL-6	4.80 ± 0.74	2.71 ± 0.27	0.60 ± 0.05
IL-12p70	0.10 ± 0.01	0.17 ± 0.02	0.18 ± 0.05
IL-1β	Below LOD	88.51 ± 3.71	4.57 ± 0.57
TNF-α	0.57 ± 0.03	0.35 ± 0.02	0.16 ± 0.01
IFN-γ	0.019 ± 0.001	0.014 ± 0.004	Below LOD

Table S8. Dilution Linearity for efMOSAIC assays of IL-12p70 in human plasma samples

Dilution Factor	Plasma 1		Plasma 2		Plasma 3	
	Dilution Corrected Conc. (pg/mL)	% Linearity	Dilution Corrected Conc. (pg/mL)	% Linearity	Dilution Corrected Conc. (pg/mL)	% Linearity
2	0.08 ± 0.02	-	0.22 ± 0.02	-	0.10 ± 0.04	-
4	0.10 ± 0.02	75	0.23 ± 0.04	95	0.10 ± 0.05	96
6	0.10 ± 0.04	99	0.14 ± 0.01	162	0.09 ± 0.07	111
8	0.12 ± 0.05	90	0.14 ± 0.04	103	0.14 ± 0.09	63
12	Below LOD	-	0.13 ± 0.09	106	0.29 ± 0.05	49
16	Below LOD	-	Below LOD	-	0.35 ± 0.19	81
24	Below LOD	-	Below LOD	-	Below LOD	-
32	Below LOD	-	Below LOD	-	Below LOD	-

Table S9. Dilution Linearity for efMOSAIC assays of IL-8 in human plasma samples

Dilution Factor	Plasma 1		Plasma 2		Plasma 3	
	Dilution Corrected Conc. (pg/mL)	% Linearity	Dilution Corrected Conc. (pg/mL)	% Linearity	Dilution Corrected Conc. (pg/mL)	% Linearity
2	9.92 ± 2.73	-	6.60 ± 1.53	-	13.29 ± 0.45	-
4	14.78 ± 2.75	67	10.69 ± 1.70	62	18.75 ± 0.79	71
6	16.69 ± 2.33	89	12.58 ± 1.59	85	20.15 ± 0.60	93
8	17.68 ± 1.12	94	11.21 ± 0.41	112	20.93 ± 0.38	96
12	19.59 ± 3.27	90	11.41 ± 2.17	98	20.43 ± 1.11	102
16	21.55 ± 1.06	91	13.79 ± 0.78	83	17.97 ± 3.43	114
24	23.93 ± 0.71	90	14.04 ± 0.86	98	19.08 ± 3.68	94
32	25.70 ± 2.07	93	15.35 ± 2.18	91	22.13 ± 1.27	86

Table S10. Dilution Linearity for efMOSAIC assays of IL-10 in human plasma samples

Dilution Factor	Plasma 1		Plasma 2		Plasma 3	
	Dilution Corrected Conc. (pg/mL)	% Linearity	Dilution Corrected Conc. (pg/mL)	% Linearity	Dilution Corrected Conc. (pg/mL)	% Linearity
2	0.11 ± 0.01	-	0.23 ± 0.04	-	0.07 ± 0.03	-
4	0.10 ± 0.01	103	0.21 ± 0.04	108	0.09 ± 0.04	82
6	0.11 ± 0.01	96	0.19 ± 0.02	111	0.07 ± 0.05	123
8	0.14 ± 0.02	78	0.23 ± 0.02	83	0.11 ± 0.09	65
12	0.13 ± 0.06	105	0.18 ± 0.04	130	0.07 ± 0.03	156
16	0.15 ± 0.01	90	0.18 ± 0.09	97	0.12 ± 0.09	57
24	0.13 ± 0.04	112	Below LOD	-	Below LOD	-
32	0.15 ± 0.11	90	Below LOD	-	Below LOD	-

Table S11. Dilution Linearity for efMOSAIC assays of IL-1β in human plasma samples

Dilution Factor	Plasma 1		Plasma 2		Plasma 3	
	Dilution Corrected Conc. (pg/mL)	% Linearity	Dilution Corrected Conc. (pg/mL)	% Linearity	Dilution Corrected Conc. (pg/mL)	% Linearity
2	Below LOD	-	48.35 ± 10.06	-	6.21 ± 0.66	-
4	Below LOD	-	90.98 ± 4.73	53	5.62 ± 1.37	110
6	Below LOD	-	98.98 ± 9.92	92	3.99 ± 1.26	141
8	Below LOD	-	102.4 ± 3.61	97	3.27 ± 0.77	122
12	Below LOD	-	75.61 ± 3.48	135	3.73 ± 4.25	88
16	Below LOD	-	80.53 ± 6.87	94	Below LOD	-
24	Below LOD	-	88.90 ± 5.85	91	Below LOD	-
32	Below LOD	-	48.35 ± 10.06	-	Below LOD	-

Table S12. Dilution Linearity for efMOSAIC assays of TNF-α in human plasma samples

Dilution Factor	Plasma 1		Plasma 2		Plasma 3	
	Dilution Corrected Conc. (pg/mL)	% Linearity	Dilution Corrected Conc. (pg/mL)	% Linearity	Dilution Corrected Conc. (pg/mL)	% Linearity
2	0.50 ± 0.05	-	0.29 ± 0.02	-	0.17 ± 0.03	-
4	0.56 ± 0.04	89	0.38 ± 0.06	76	0.15 ± 0.06	115
6	0.54 ± 0.09	104	0.33 ± 0.03	115	0.17 ± 0.05	89
8	0.54 ± 0.03	101	0.35 ± 0.06	94	0.14 ± 0.01	121
12	0.60 ± 0.26	90	0.41 ± 0.12	85	0.16 ± 0.08	88
16	0.68 ± 0.12	88	Below LOD	-	Below LOD	-
24	Below LOD	-	Below LOD	-	Below LOD	-
32	Below LOD	-	Below LOD	-	Below LOD	-

Table S13. Dilution Linearity for efMOSAIC assays of IL-6 in human plasma samples

Dilution Factor	Plasma 1		Plasma 2		Plasma 3	
	Dilution Corrected Conc. (pg/mL)	% Linearity	Dilution Corrected Conc. (pg/mL)	% Linearity	Dilution Corrected Conc. (pg/mL)	% Linearity
2	2.05 ± 0.12	-	3.64 ± 0.61	-	0.78 ± 0.08	-
4	3.11 ± 0.43	66	3.61 ± 0.34	101	0.60 ± 0.32	128
6	3.80 ± 0.29	82	3.05 ± 0.40	118	0.54 ± 0.09	111
8	4.42 ± 0.07	86	3.30 ± 0.40	92	0.58 ± 0.10	95
12	6.94 ± 0.50	64	1.95 ± 0.84	169	0.49 ± 0.13	117
16	6.37 ± 0.59	109	1.88 ± 0.53	104	Below LOD	-
24	6.89 ± 0.68	92	1.87 ± 1.87	100	Below LOD	-
32	Below LOD	-	2.39 ± 0.56	78	Below LOD	-

Table S14. Dilution Linearity for efMOSAIC assays of IFN-γ in human plasma samples

Dilution Factor	Plasma 1		Plasma 2		Plasma 3	
	Dilution Corrected Conc. (pg/mL)	% Linearity	Dilution Corrected Conc. (pg/mL)	% Linearity	Dilution Corrected Conc. (pg/mL)	% Linearity
2	0.015 ± 0.008	-	0.023 ± 0.004	-	Below LOD	-
4	0.017 ± 0.007	86	0.017 ± 0.005	137	Below LOD	-
6	0.017 ± 0.010	99	0.009 ± 0.003	189	Below LOD	-
8	0.022 ± 0.016	80	0.006 ± 0.002	154	Below LOD	-
12	0.017 ± 0.014	124	Below LOD	-	Below LOD	-
16	0.022 ± 0.013	81	Below LOD	-	Below LOD	-
24	0.019 ± 0.015	116	Below LOD	-	Below LOD	-
32	Below LOD	-	Below LOD	-	Below LOD	-

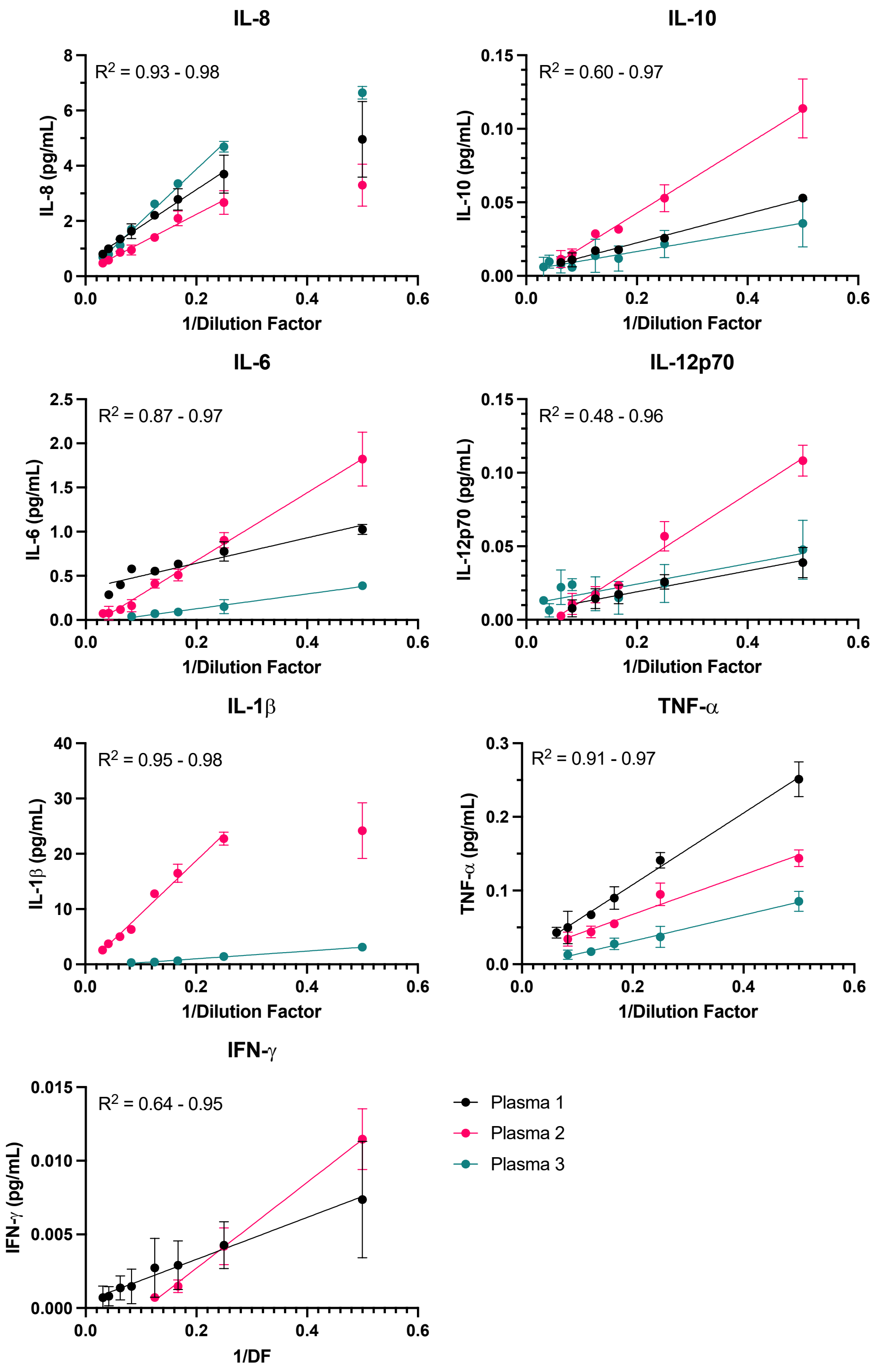

Figure S25. Dilution linearity of proteins in healthy human plasma from three different individuals. Error bars are standard deviation from three replicates for samples and blanks, and curves were fitted using simple linear regression model.

Given that efMOSAIC can accurately measure endogenous proteins in human plasma samples by parallelism, we continued assay validation by spike and recovery of recombinant protein. For each set of assays, a spike was added to 8× diluted plasma at one of three different concentrations that represented low, medium, and high amounts of endogenous protein. Separately, a sample of spike in buffer was measured as a control. An assay had promising recovery if the measured spike concentration was between 80 – 120 % of the expected spiked amount (Equation S2). A summary of the experimental findings (Table S14) suggests that recovery for most proteins falls within the ideal range however, IFN-γ did have unexpectedly low recovery in Plasma 3. We believe that bead aggregation effects may have contributed to a lower recovery for this assay and we are currently exploring new strategies for minimizing bead loss during sample measurement.

Equation S2.
$$\% \textbf{ Recovery of Spiked Protein} = \frac{[C_{Observed}]-[C_{Endogenous}]}{[C_{Spiked}]} \times \mathbf{100}\ \%$$

Table S15. Validation of efMOSAIC assay with Spiked Recovery of Proteins in Individual Human Plasma Samples.

Singleplex efMOSAIC Assays	Amount of Spiked Protein (pg/mL)	Percent Recovery of Spiked Protein (%)		
		Plasma 1	Plasma 2	Plasma 3
IL-8	4	117 ± 13	93 ± 37	115 ± 25
	8	97 ± 34	120 ± 19	121 ± 42
	10	98 ± 21	91 ± 36	63 ± 45
IL-10	0.2	82 ± 16	69 ± 24	80 ± 25
	2	75 ± 16	91 ± 3	72 ± 28
	5	74 ± 4	59 ± 43	73 ± 9
IL-6	1	84 ± 19	68 ± 12	95 ± 7
	4	70 ± 20	65 ± 7	98 ± 23
	10	72 ± 32	70 ± 5	109 ± 20
IL-12p70	0.2	87 ± 19	73 ± 18	75 ± 12
	1	76 ± 15	104 ± 31	81 ± 20
	10	68 ± 27	103 ± 28	85 ± 23
IL-1β	10	92 ± 11	55 ± 30	58 ± 17
	20	130 ± 8	89 ± 30	91 ± 8
	50	129 ± 13	97 ± 13	97 ± 4
TNF-α	0.2	81 ± 9	65 ± 14	82 ± 11
	0.5	106 ± 5	89 ± 23	105 ± 7
	1	90 ± 2	96 ± 7	95 ± 2
IFN-γ	0.5	71 ± 1	66 ± 5	28 ± 2
	1	63 ± 2	62 ± 4	29 ± 2
	5	74 ± 1	68 ± 1	31 ± 2

Multiplexed Simoa Assays

The Simoa calibration curves and protein dropout assays for the multiplexed detection of IL-10 and TNF-α were acquired on three different days, Figure S25 – S27, Table S14.

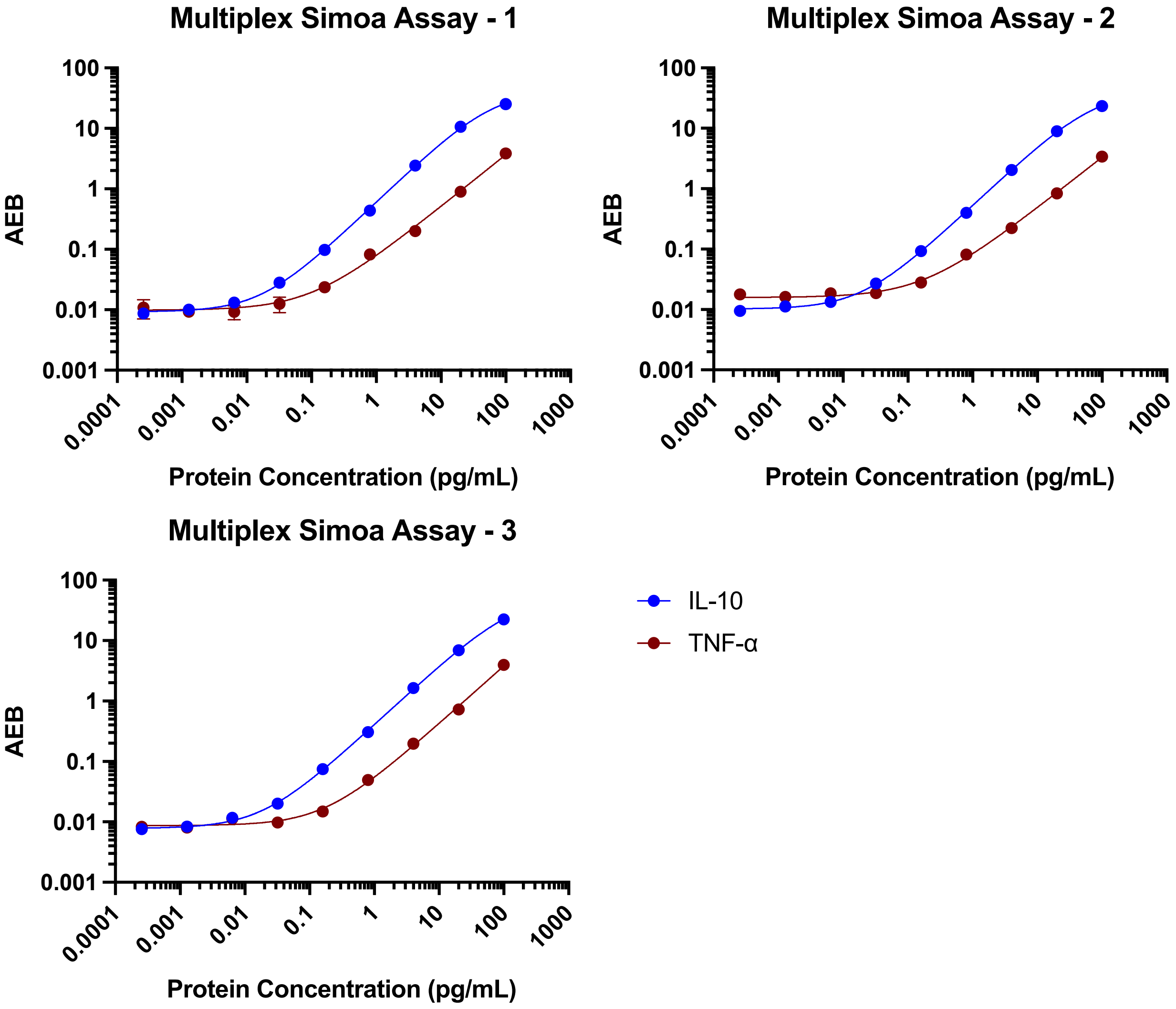

Figure S26. Simoa calibration curves for IL-10 and TNF-α multiplexed assays were done in sample diluent using both protein calibrators. Replicate measurements were made on three different days and error bars were standard deviation from three replicates for samples and blanks. Curves were fitted using the four-parameter logistic regression (4PL).

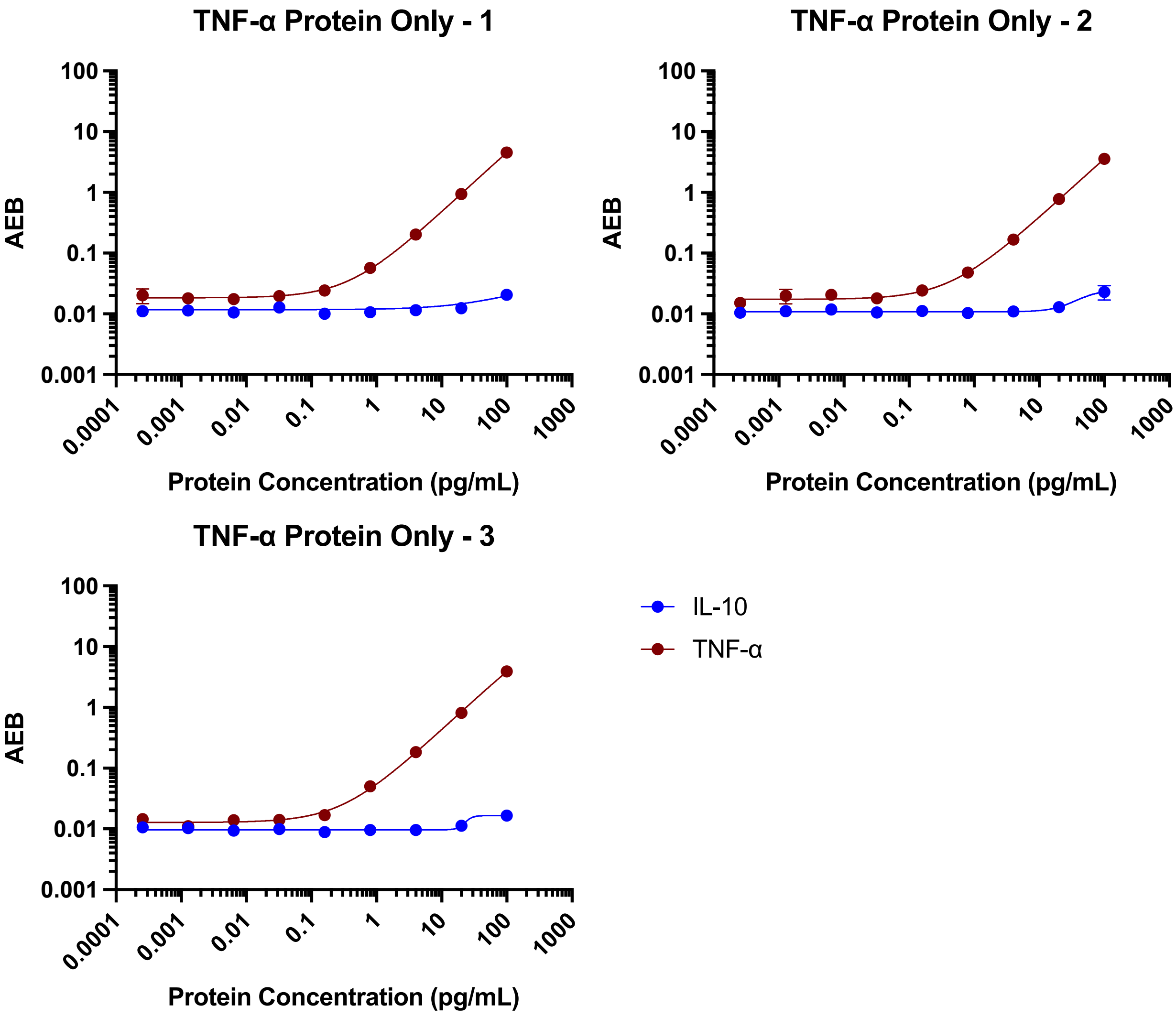

Figure S27. Simoa calibration curves for IL-10 and TNF-α multiplexed assays were done in sample diluent using only TNF-α protein calibrators (IL-10 dropout). Replicate measurements were made on three different days and error bars were standard deviation from three replicates for samples and blanks. Curves were fitted using the four-parameter logistic regression (4PL).

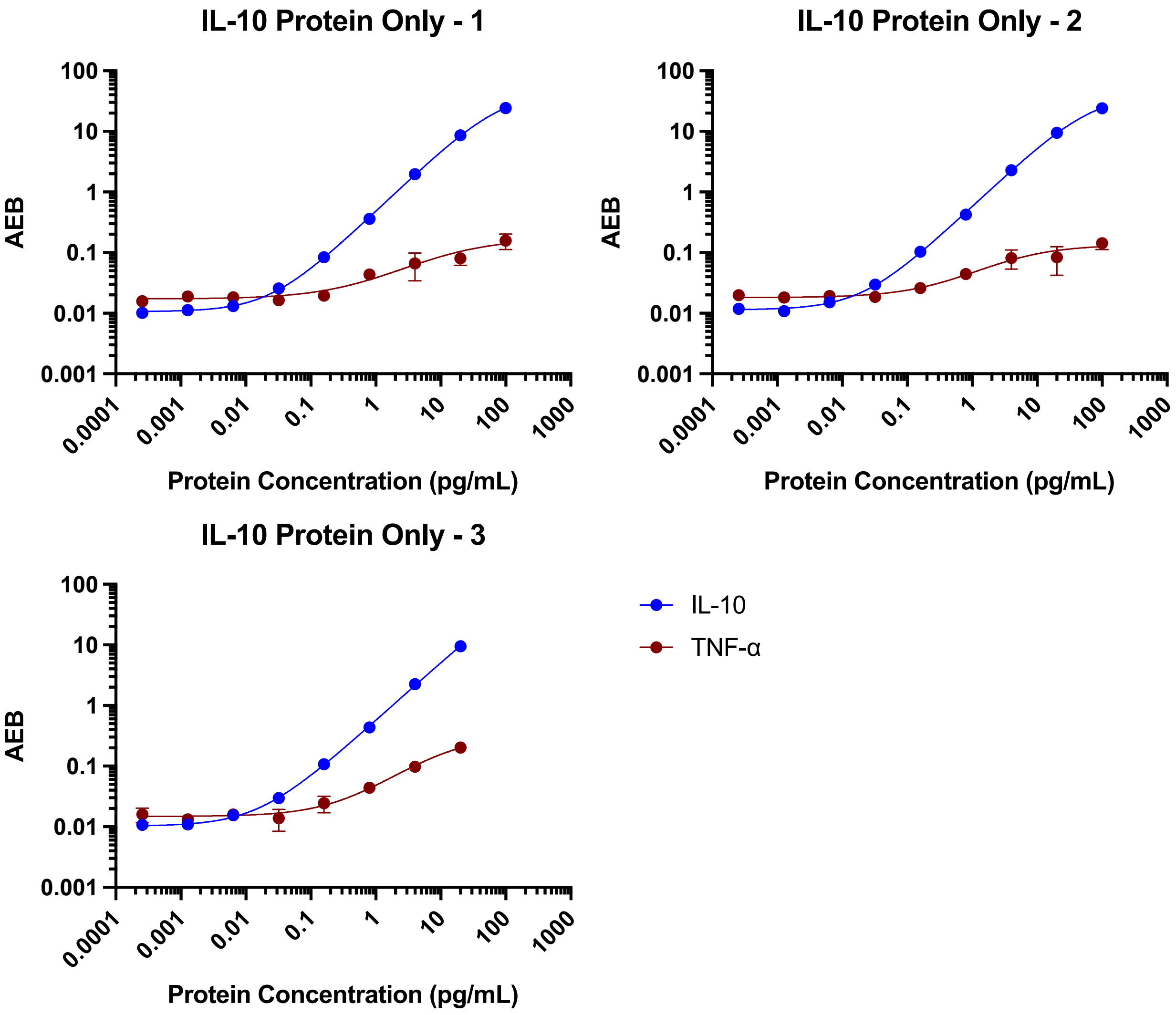

Figure S28. Simoa calibration curves for IL-10 and TNF-α multiplexed assays were done in sample diluent using only IL-10 protein calibrators (TNF-α dropout). Replicate measurements were made on three different days and error bars were standard deviation from three replicates for samples and blanks. Curves were fitted using the four-parameter logistic regression (4PL).

Table S16. Summary of the LODs and LLOQs for the Duplex Simoa Assay

Assay Format	Bead Channel	Limit of Detection (fg/mL)*				Lower Limit of Quantification (fg/mL)*			
		1	2	3	Average	1	2	3	Average
Multiplex Both Proteins	IL-10	2	3	9	5 ± 4	7	9	30	15 ± 13
	TNF-α	25	1	134	53 ± 71	62	35	479	$(2.0 \pm 2.5) \times 10^2$
Protein Dropout									
TNF-α only	IL-10	-	-	-	-	-	-	-	-
	TNF-α	433	79	384	$(3 \pm 2) \times 10^2$	1419	433	1269	$(1.0 \pm 0.5) \times 10^3$
IL-10 only	IL-10	5	3	1	3 ± 2	14	9	6	10 ± 4
	**TNF-α****	**100**	**13**	**202**	**$(1.0 \pm 1.0) \times 10^3$**	**278**	**77**	**785**	**$(3.8 \pm 3.7) \times 10^2$**

*LOD and LLOQ are measured as 3 and 10 SD above noise, respectively. Each set of replicates was done of a different day. ** We observed significant signal crosstalk in the TNF-α (750) color channel in protein dropout experiments.

Multiplexed efMOSAIC Assays

The efMOSAIC calibration curves and protein dropout assays for the multiplexed detection of IL-10 and TNF-α were acquired on three different days, Figure S28 – S30, Table S15.

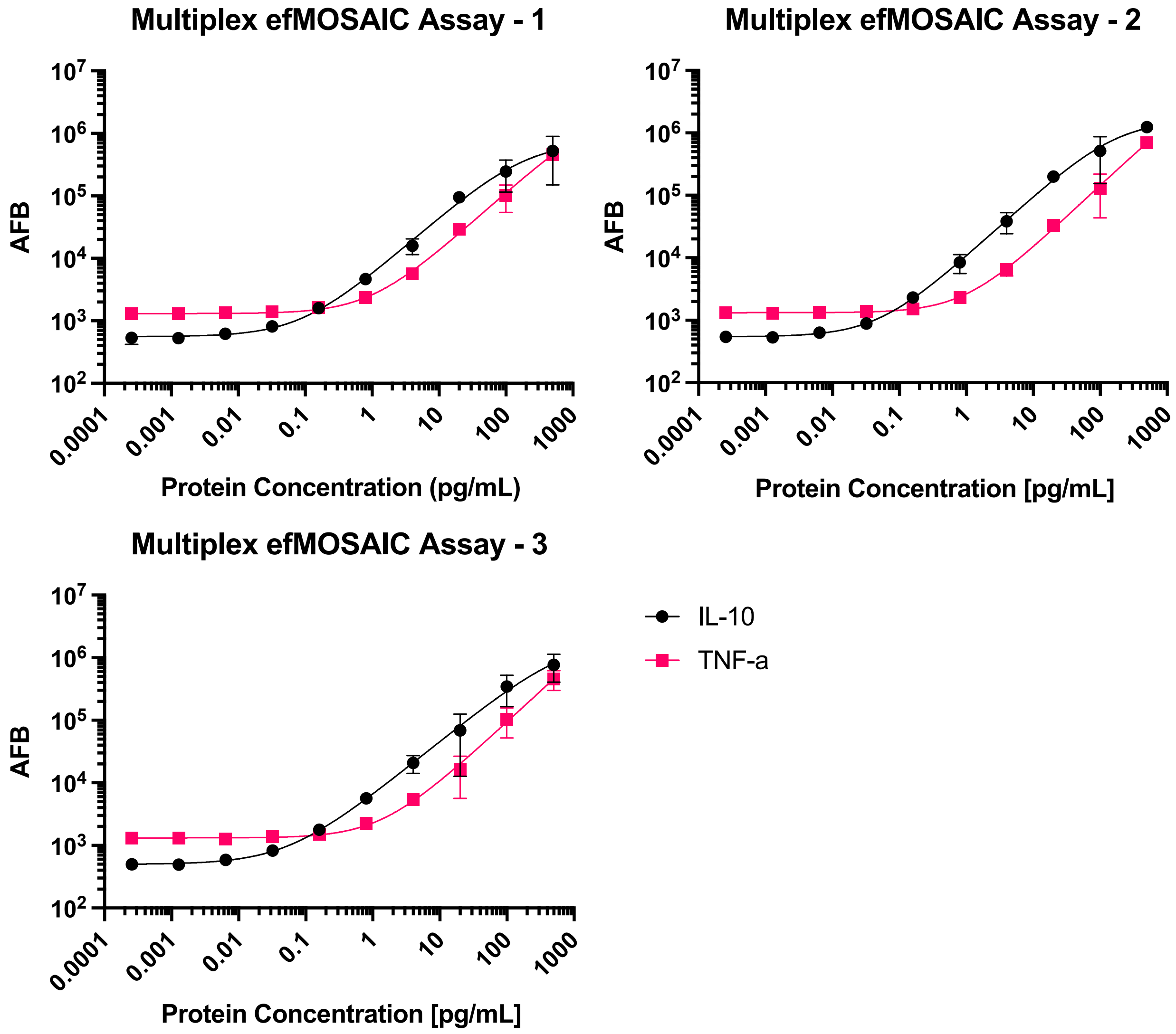

Figure S29. efMOSAIC calibration curves for IL-10 and TNF-α multiplexed assays were done in sample diluent using both protein calibrators. Replicate measurements were made on three different days and error bars were standard deviation from three replicates for samples and blanks. Curves were fitted using the four-parameter logistic regression (4PL).

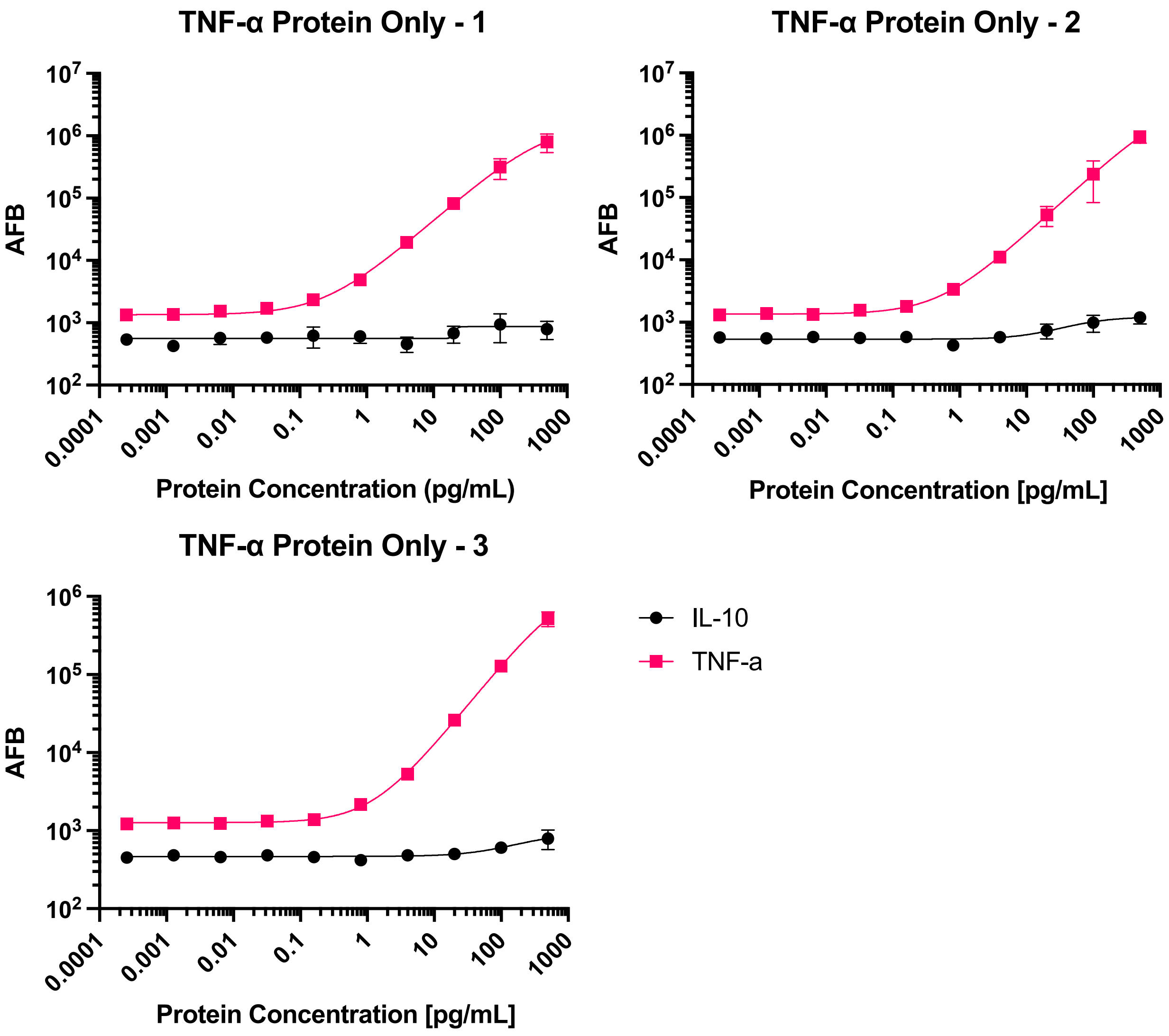

Figure S30. efMOSAIC calibration curves for IL-10 and TNF-α multiplexed assays were done in sample diluent using only TNF-α protein calibrators (IL-10 dropout). Replicate measurements were made on three different days and error bars were standard deviation from three replicates for samples and blanks. Curves were fitted using the four-parameter logistic regression (4PL).

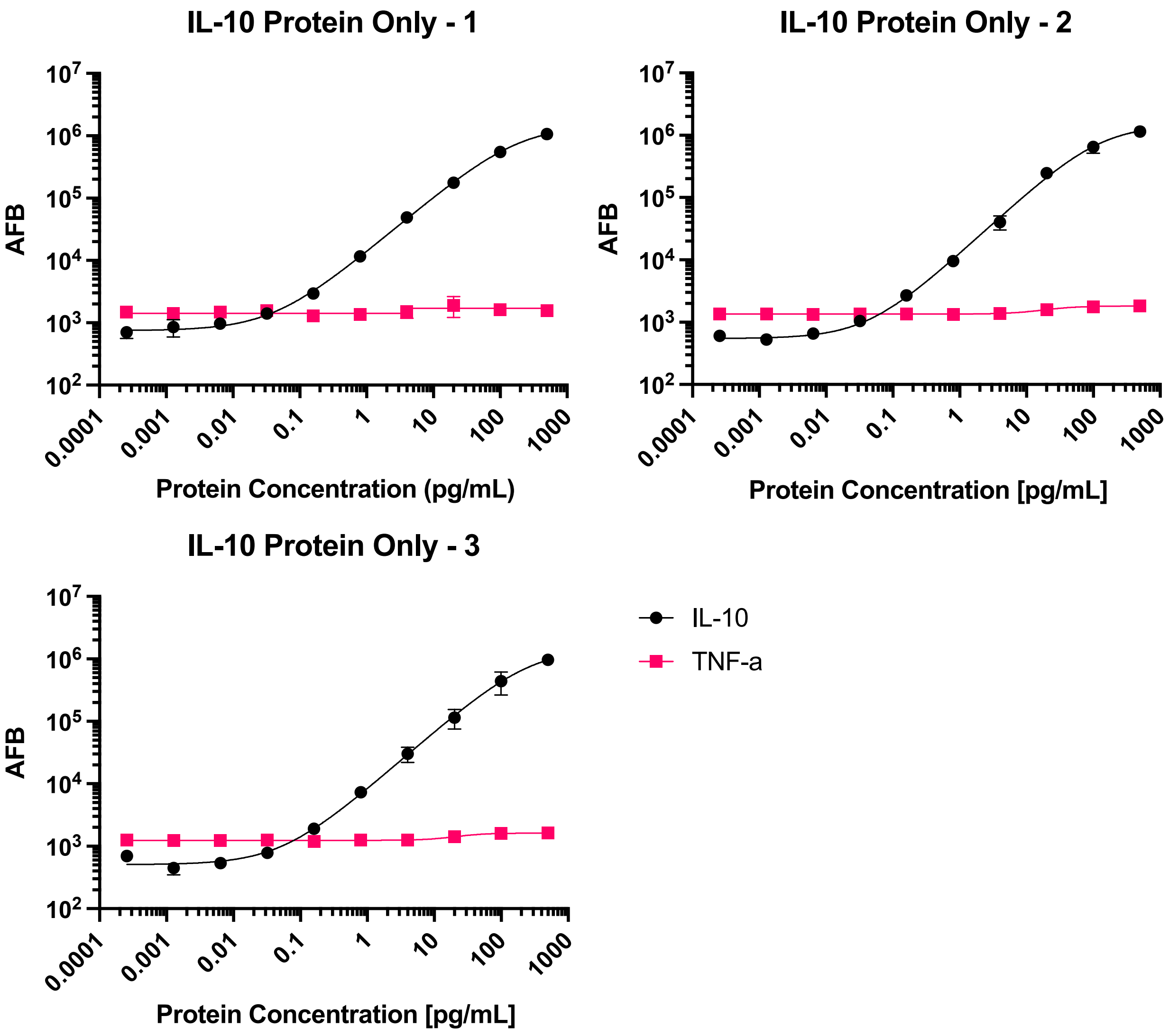

Figure S31. efMOSAIC calibration curves for IL-10 and TNF-α multiplexed assays were done in sample diluent using only IL-10 protein calibrators (TNF-α dropout). Replicate measurements were made on three different days and error bars were standard deviation from three replicates for samples and blanks. Curves were fitted using the four-parameter logistic regression (4PL).

Table S17. Summary of the LODs and LLOQs for the Duplex efMOSAIC Assay

Assay Format	Bead Channel	Limit of Detection (fg/mL)*				Lower Limit of Quantification (fg/mL)*			
		1	2	3	Average	1	2	3	Average
Multiplex Both Proteins	IL-10	44	16	12	24 ± 17	155	52	46	84 ± 61
	TNF-α	238	69	129	$(1.5 \pm 0.9) \times 10^2$	910	203	489	$(5.3 \pm 3.6) \times 10^2$
Protein Dropout									
TNF-α only	IL-10	**-**	**9.3×10^3**	**6.3×10^4**	**$(3.6 \pm 3.8) \times 10^4$**	**-**	**6.2×10^4**	**3.2×10^5**	**$(1.9 \pm 1.9) \times 10^5$**
	TNF-α	13	51	134	66 ± 62	115	209	377	234 ± 133
IL-10 only	IL-10	5	9	8	7 ± 2	24	37	37	33 ± 8
	**TNF-α****	**-**	**5.4×10^3**	**1.3×10^4**	**$(9.2 \pm 5.4) \times 10^3$**	**-**	**1.7×10^4**	**5.3×10^4**	**$(3.5 \pm 2.5) \times 10^4$**

*LOD and LLOQ are measured as 3 and 10 SD above noise, respectively. Each set of replicates was done of a different day. ** We observed some signal crosstalk in the IL-10 (488) and TNF-α (750) color channel in protein dropout experiments. However, when compared to the Simoa assays, these crosstalk signals were not significant.

Photobleaching Dye-encoded Beads Can Improve Signal/Background Ratio

Previous research has demonstrated that photobleaching the autofluorescence of magnetic beads enhances the signal-to-background ratio (SBR) and sensitivity of bead-based assays.[72] In this study, we demonstrate the applicability of this method to color-coded magnetic beads utilized in the efMOSAIC method, aiming to reduce background and enhance the SBR. The Simoa 750 beads are labeled with HiLyte™ Fluor 750, which possesses a broad emission spectrum (AAT Bioquest spectra viewer). Consequently, these labeled beads emit fluorescence in any channel that detects part of their emission, such as the 647-reporter (APC-A) channel. This phenomenon accounts for the observed signal in the 647-reporter channel even at zero analyte concentration. Photobleaching resulted in a 79% reduction in this background signal (Figure S31) and a ~3-fold improvement in the SBR (Figure S32). Although the signal in the 750 beads channel (APCA750-A) was reduced by 94%, it remained detectable. In contrast, Simoa 488 beads, labeled with Alexa Fluor 488, exhibit negligible emission (less than 0.01% of the AF488 peak intensity) at 660 nm (AAT Bioquest spectra viewer), resulting in a lower background at zero analyte concentration compared to the 750 beads (Figure S32). Post-photobleaching, the background of the 750 beads was comparable to that of unbleached 488 beads (Figure S32a). This technique is applicable to any assay developed on the efMOSAIC platform, offering users an additional method to mitigate background.

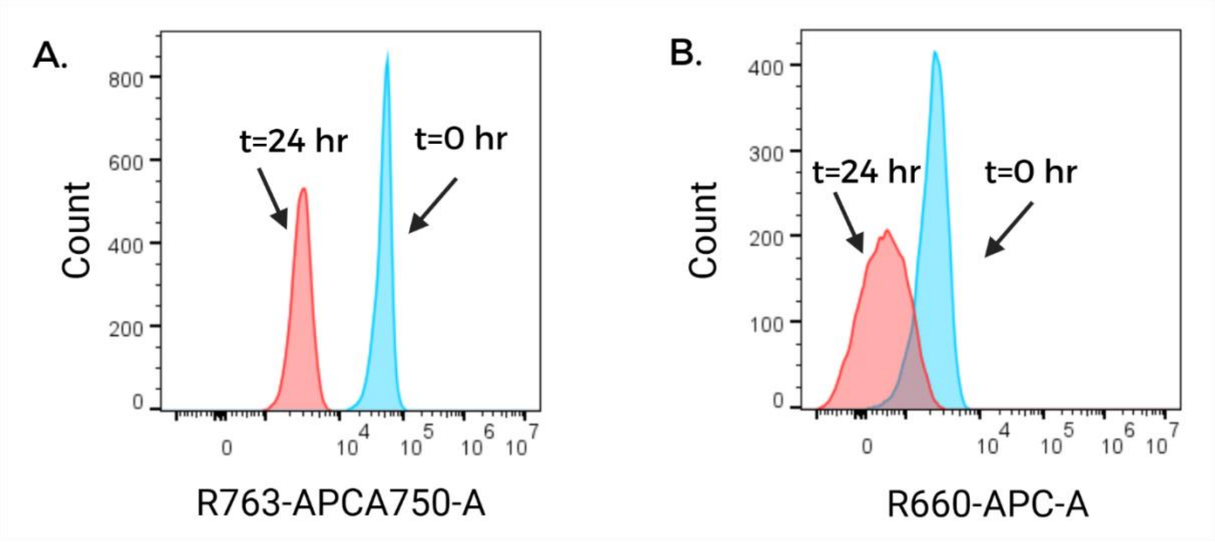

Figure S32. Conjugated 750-TNF-α magnetic beads MFI before (t=0 hr) and after (t=24 hr) photobleaching in the (A) 750 channel, and in the (B) 647 (APC) channel. There is a fluorescence reduction of 94% and 79% from the initial MFI in the 750 and 647 channels, respectively.

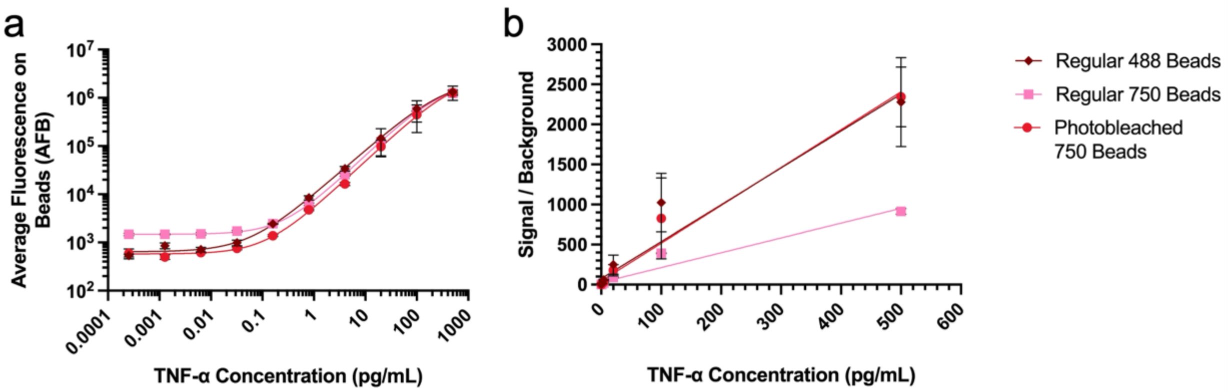

Figure S33. The calibration curves of dye-encoded and photbleached beads. (a) Calibration curves for singleplex assays of TNF-α using 488, 750, and photobleached 750 beads. Error bars were standard deviation from three replicates for samples and blanks. Curves were fitted using the four-parameter logistic regression (4PL). (b) Complementary analysis of the signal/background ratio for singleplex TNF-α assays. Error bars were standard deviation from three replicates for samples and blanks. Curves were fitted using simple linear regression model.

Reagents and Consumables

Bead conjugation buffer (#101357), bead wash buffer (#101355), bead blocking buffer (#101356), bead diluent buffer (#101362), homebrew detector and sample diluent (#101359), biotinylation buffer (#101358), system wash buffer 1 (#100486), 2.7 μm carboxylated paramagnetic beads (#104006 and #103529) were from Quanterix Corporation (Billerica, MA, USA).

Ultrapure™ SSC 20× (#15557044), Ultrapure™ DNase/RNase-free Distilled Water (#10977015), PBS pH 7.4 (#10010023), Ultrapure™ Agarose (#16500100), 1 Kb Plus DNA Ladder (#10787018), 100 bp DNA Ladder (#15628019), Tween™20 Surface-Amps™ Detergent Solution (#85114), Triton X-100 for molecular biology (#T8787), $MgCl_2$ 1M solution (#AM9530G), EZ-Link™ NHS-PEG4-Biotin No-Weigh™ format (#A39259), Pierce™ EDC No-Weigh™ format (A35391), SYBR gold nucleic acid gel stain (#S11494) were from ThermoFisher Scientific (Waltham, MA, USA).

Streptavidin (#189730), Dibenzocyclooctyne-PEG4-*N*-hydroxysuccinimidyl ester ≥90% (DBCO-PEG4-NHS; #764019), Dimethyl sulfoxide (DMSO; #D8414), Amicon® Ultra Centrifugal Filter 50 kDa MWCO (#UFC5050), Bovine Serum Albumin (#A9418), Sodium Azide (#S2002), Dextran Sulfate 50% (w/v) solution (#S4030) were from MilliporeSigma (Burlington, MA, USA).

Microplate, 96 Well, PS, F-bottom, Chimney Well μ-clear®, black, med. Binding (#655096) and Microplate, 96 Well, PS, F-bottom, Chimney Well μ-clear®, black, non. Binding, (#655096) were from Greiner Bio-One (Monroe, NC, USA).

Capture and detector antibodies (see Table S18 for full list) were from Biolegend (San Diego, CA, USA), BD Biosciences (Franklin Lakes, NJ, USA), Abcam (Waltham, MA, USA), or R&D Systems (Minneapolis, MN, USA).

Synthetic nucleic acids were from Integrated DNA Technologies, IDT (Coralville, IA, USA).

Recombinant proteins were from R&D Systems (Minneapolis, MN, USA).

Synthetic Oligonucleotides

All oligonucleotides used in this report were synthesized by Integrated DNA Technologies (IDT), Table S16. Unlabeled oligonucleotides were custom synthesized and purified with standard desalting. ATTO647NN, ATTO565N, Alexa647N, and AzideN (N_3) labelled oligonucleotides were HPLC purified. Nucleic acid sequences were dissolved in ultrapure water to a concentration of 100 µM, and stored at –20°C.

Table S18. Oligonucleotide Sequences and Modifications for the efMOSAIC Platform

Oligonucleotide ID	Oligonucleotide Sequences (5' → 3')
Unlabelled Oligonucleotides	
Initiator Basic	GCTCGACGTTCCTTTGCAACA
H1 Basic	TGTTGCAAAGGAACGTCGAGC**TGTAATGGT**GCTCGACGTTCC
H2 Basic	GCTCGACGTTCC**TTTGCAACA**GGAACGTCGAGCACCATTACA
Modified Oligonucleotides	
Initiator Azide	/5AzideN/TTTTTTTTTTTTTTTGCTCGACGTTCCTTTGCAACA
H1-ATTO647 (5' mod)	/5ATTO647NN/TGTTGCAAAGGAACGTCGAGC**TGTAATGGT**GCTCGACGTTCC
H2-ATTO647 (5' mod)	/5ATTO647NN/GCTCGACGTTCC**TTTGCAACA**GGAACGTCGAGCACCATTACA
H1-ATTO647 (3' mod)	TGTTGCAAAGGAACGTCGAGC**TGTAATGGT**GCTCGACGTTCC/3ATTO647NN/
H2-ATTO647 (3' mod)	GCTCGACGTTCC**TTTGCAACA**GGAACGTCGAGCACCATTACA/3ATTO647NN/
H1-Alexa647 (5' mod)	/5Alex647N/TGTTGCAAAGGAACGTCGAGC**TGTAATGGT**GCTCGACGTTCC
H2-Alexa647 (5' mod)	/5Alex647N/GCTCGACGTTCC**TTTGCAACA**GGAACGTCGAGCACCATTACA

*Underlined bases denote the toehold region, and **bolded bases** represent the hairpin.

Antibodies and Recombinant Proteins

A list of the suppliers and lot numbers for antibodies and protein standards is given in Table S17.

Table S19. Capture Antibodies, Detector Antibodies, and Recombinant Protein Standards

Protein Targets	Capture Antibody	Recombinant Protein	Detector Antibody
IL-1β	508202 (Biolegend)<br>Lot: B366264	201-LB-005 (R&D Systems)<br>Lot: AD2022032	511704 (Biolegend)<br>Lot: B343977
IL-10	506802 (Biolegend)<br>Lot: B360597	217-IL-005 (R&D Systems)<br>Lot: ET3321101	501501 (Biolegend)<br>Lot: B376499
IL-8	554716 (BD Biosciences)<br>Lot: 3164008	208-IL-010 (R&D Systems)<br>Lot: DILS0322072	554718 (BD Biosciences)<br>Lot: 0286237
IL-12p70	511002 (Biolegend)<br>Lot: B373080	219-IL-005 (R&D Systems)<br>Lot: PY2722111	508801 (Biolegend)<br>Lot: B363580
TNF-α	MAB610 (R&D Systems)<br>Lot: AVR2822061	210-TA (R&D Systems)<br>Lot: DDHB0422043	ab9635 (Abcam)<br>Lot: GR3180307-24
IL-6	MAB206 (R&D Systems)<br>Lot: HD5422051	206-IL-010 (R&D Systems)<br>Lot: OJZ2222092	BAF206 (R&D Systems)<br>Lot: SV3223041
IFN-γ	507502 (Biolegend)<br>Lot: B336636	285-IF-100 (R&D Systems)<br>Lot: RAX2323021	MAB285 (R&D Systems)<br>Lot: KW2021052

Buffers Recipes for the efMOSAIC assay

Amplification Buffer

The amplification buffer used to prepare the Streptavidin Signal Amplifier (SSA) reagent was 5 × SSC + 0.1 % Tween20 + 10 % w/v Dextran Sulfate + 0.1 M $MgCl_2$ + 0.02 % Sodium Azide. To prepare a 40 mL solution, mix the following reagents in a 50 mL centrifuge tube:

- 10 mL of 20 × SSC (sodium chloride sodium citrate solution).
- 0.4 mL of Tween20 (from a 10 % w/v solution).
- 8 mL of 50 % w/v Dextran Sulfate.
- 4 mL of $MgCl_2$ solution [1M stock solution].
- 0.16 mL of 5 % Sodium Azide Solution.
- 17.44 mL of Ultrapure™ water.

Wash Buffer

The wash buffer used in efMOSAIC assays was 1 × SSC + 0.1 % Tween20. For a 500 mL solution, mix the following reagents in a bottle:

- 25 mL of 20 × SSC (sodium chloride sodium citrate solution).
- 5 mL of Tween20 (10 % w/v solution).
- 470 mL of Ultrapure™ water.

Summary of efMOSAIC and Simoa Assay Conditions

For both the singleplex and multiplex formats of Simoa assays, 150 pM of SBG was used unless stated otherwise. For multiplexed Simoa assays, unbleached 750 beads were used in all experiments. For efMOSAIC multiplexed assays, both photobleached and unbleached 750 beads were investigated, Table S18.

Table S20. Experimental Parameters for the efMOSAIC and Simoa Assays

Analyte	efMOSAIC				SIMOA			
	Assay Bead Number	Detector Conc. (μg/mL)	Assay Type	Incubation times (min)	Assay Bead Number	Detector Conc. (μg/mL)	Assay Type	Incubation times (min)
Singleplex Assays								
IL-10	20,000	2.25	3-step	60-15-30	500,000	0.30	2-step with 25 μL RGP	35-5
IL-12p70	20,000	2.25		60-15-30	500,000	0.30		35-5
IL-8	20,000	2.25		60-15-15	500,000	0.15		35-5
IL-6	20,000	2.25		60-15-30	500,000	0.30		35-5
IL-1b	20,000	2.25		60-15-15	500,000	0.60		35-5
TNF-a	20,000	2.25		60-15-30	500,000	0.30		35-5
IFN-y	20,000	2.25		60-15-30	500,000	0.30		35-5
Multiplex Assay								
IL-10 (488 beads)	20,000	2.25	3-step	60-15-30	250,000	0.30	2-step with 25 μL RGP	35-5
TNF-a (750 beads)	20,000	2.25			250,000	0.30		35-5

Summary of efMOSAIC Assay Workflow

The standard efMOSAIC assay was done in a 3-step format as highlighted by the three incubation steps of beads with sample, detector antibodies, and signal amplification reagent, Table S19. Some variations in these parameters have been investigated however, it can affect the overall performance of efMOSAIC assays, Figure S7, Table S1.

Table S21. efMOSAIC Assay Workflow

Steps	efMOSAIC Workflow	Time (mins)
1	Incubation of beads and samples (Step 1)	60
2	Six cycles of washing on the plate washer	10
3	Incubation with biotinylated detector antibodies (Step 2)	15
4	Six cycles of washing on the plate washer	10
5	Incubation with the streptavidin-conjugated signal amplification reagent (Step 3)	15 to 30
6	Manual washing with final wash buffer	10
7	Load onto flow cytometer and start measurement	N/A
	Total Time:	120 to 135
